# Inpatient-level care at home delivered by virtual wards and hospital at home: a systematic review and meta-analysis of complex interventions and their components

**DOI:** 10.1186/s12916-024-03312-3

**Published:** 2024-04-02

**Authors:** Chunhu Shi, Jo Dumville, Fernando Rubinstein, Gill Norman, Akbar Ullah, Saima Bashir, Peter Bower, Emma R. L. C. Vardy

**Affiliations:** 1 School of Health Sciences, Faculty of Biology, Medicine and Health, Manchester Academic Health Science Centre, University of Manchester, Manchester, UK; 2 NIHR Applied Research Collaboration Greater Manchester (ARC-GM), Manchester, UK; 3 Centre for Primary Care and Health Services Research, University of Manchester, Manchester, UK; 4 NIHR School for Primary Care Research, Manchester Academic Health Science Centre, Division of Population Health, Health Services Research and Primary Care, University of Manchester, Manchester, UK; 5 Evidence Synthesis Group, Population Health Sciences Institute, Newcastle University, Newcastle Upon Tyne, UK; 6 NIHR Innovation Observatory, Population Health Sciences Institute, Newcastle University, Newcastle Upon Tyne, UK; 7 Manchester Centre for Health Economics, Faculty of Biology Medicine and Health, The University of Manchester, Manchester, UK; 8 Oldham Care Organisation, Northern Care Alliance NHS Foundation Trust, Oldham, UK

**Keywords:** Virtual ward, Hospital at home, Telemedicine, Systematic review

## Abstract

**Background:**

Technology-enabled inpatient-level care at home services, such as virtual wards and hospital at home, are being rapidly implemented. This is the first systematic review to link the components of these service delivery innovations to evidence of effectiveness to explore implications for practice and research.

**Methods:**

For this review (registered here https://osf.io/je39y), we searched Cochrane-recommended multiple databases up to 30 November 2022 and additional resources for randomised and non-randomised studies that compared technology-enabled inpatient-level care at home with hospital-based inpatient care. We classified interventions into care model groups using three key components: clinical activities, workforce, and technology. We synthesised evidence by these groups quantitatively or narratively for mortality, hospital readmissions, cost-effectiveness and length of stay.

**Results:**

We include 69 studies: 38 randomised studies (6413 participants; largely judged as low or unclear risk of bias) and 31 non-randomised studies (31,950 participants; largely judged at serious or critical risk of bias). The 69 studies described 63 interventions which formed eight model groups. Most models, regardless of using low- or high-intensity technology, may have similar or reduced hospital readmission risk compared with hospital-based inpatient care (low-certainty evidence from randomised trials). For mortality, most models had uncertain or unavailable evidence. Two exceptions were low technology-enabled models that involve hospital- and community-based professionals, they may have similar mortality risk compared with hospital-based inpatient care (low- or moderate-certainty evidence from randomised trials). Cost-effectiveness evidence is unavailable for high technology-enabled models, but sparse evidence suggests the low technology-enabled multidisciplinary care delivered by hospital-based teams appears more cost-effective than hospital-based care for those with chronic obstructive pulmonary disease (COPD) exacerbations.

**Conclusions:**

Low-certainty evidence suggests that none of technology-enabled care at home models we explored put people at higher risk of readmission compared with hospital-based care. Where limited evidence on mortality is available, there appears to be no additional risk of mortality due to use of technology-enabled at home models. It is unclear whether inpatient-level care at home using higher levels of technology confers additional benefits. Further research should focus on clearly defined interventions in high-priority populations and include comparative cost-effectiveness evaluation.

**Trial registration:**

https://osf.io/je39y.

**Supplementary Information:**

The online version contains supplementary material available at 10.1186/s12916-024-03312-3.

## Background

There are huge pressures on health systems globally; in large part due to an ageing population and a corresponding increase in demands on health care services. The COVID-19 pandemic placed additional pressure on a stretched system. In the UK National Health Service (NHS), between April 2021 and March 2022, adults aged 60 and above were in receipt of half (50.2%) of the 19.6 million NHS hospital consultant episodes recorded [1]. In the USA, those older than 65 years accounted for 36% of hospitalisations in 2017 [2]. Current inpatient provision cannot keep up with changing demographic and health care demands. Alternative service delivery models have been developed to provide inpatient-level care outside hospital settings. Such home-based inpatient care models are designed to prevent inpatient admission into hospital (*step-up* into hospital-based care) or to facilitate early discharge (*step down* from hospital-based care).

Service delivery models that offer inpatient care at home have been in use in various formats for several years. Recently, in countries including the UK, there has been increasing investment in the provision of inpatient care at home models [3]. A commonly described model is ‘virtual wards’. This term broadly refers to care services that offer a limited period of a hospital ward-level acute care at a patient’s place of residence and involve use of technology [4]. ‘Virtual wards’ entail variable face-to-face activity component and in some cases patients may be solely managed remotely. ‘Hospital at home’ is another common model and is broadly used to describe face-to-face provision of, often multidisciplinary, inpatient care at home that would otherwise be delivered in hospital [5]. As such ‘hospital at home’ could stand alone or be a component of a virtual ward.

The terms ‘virtual ward’ and ‘hospital at home’ have historically been used somewhat interchangeably, but this can complicate evidence synthesis and its interpretation. In this review, we use ‘inpatient-level care at home’ as an umbrella term for a set of complex interventions that allow people to receive inpatient-level acute care outside of hospital. Such complex interventions involve various components, including technology involvement, workforce structure, clinical activities, information and support provision, and use of information systems.

Our scoping search identified 11 published systematic reviews of interventions described as ‘hospital at home’ or ‘virtual wards’, compared with hospital-based inpatient care, in a range of populations [6]. These reviews included not only inpatient-level care at home including face-to-face delivered care models but also remote monitoring that may not involve inpatient-level care [6]. There is a lack of existing evidence on technology-enabled models that are the focus of this review. Whilst models with different components may differ in their effectiveness, [7] care at home models included in previous reviews have not been described in detail in terms of their constituent components. Clearly describing components of existing models and, where possible, linking the components to effectiveness evidence are important for informing implementation of future innovations.

### Objectives

We aimed to (1) identify and describe the components of ‘inpatient-level care at home’ models reported in comparative effectiveness research; and (2) synthesise identified research to assess the clinical, cost-effectiveness and safety of ‘inpatient-level care at home’ models, compared with hospital-based inpatient care, in people with any health conditions.

## Methods

We follow generic Cochrane Systematic Review Methodology to conduct this systematic review and meta-analysis and follow the 2020 PRISMA guideline to report it [8, 9]. The pre-registered protocol is available at the Open Science Framework (https://osf.io/je39y) [10].

### Search strategy and selection criteria

We searched Ovid MEDLINE including In-Process & Other Non-Indexed Citations (1946), Ovid Embase (1974), Cochrane Central Register of Controlled Trials (CENTRAL), and EBSCO CINAHL Plus (1937) from the inception until 30 November 2022 for English language publications. See Additional file 1: Text 1 for full search methods. We also searched the ClinicalTrials.gov and World Health Organization International Clinical Trials Registry Platform for ongoing studies; the reference lists of the two up-to-date Cochrane Reviews; [11, 12] and MedRxiv.org between 2019 and 2022 for recently completed but unpublished studies.

Using Rayyan to support study selection processes, two authors (CS, FR) independently assessed titles and abstracts for potentially eligible studies. Two review authors (CS and FR, AU or SB) then independently inspected the full-text of these potentially eligible studies. At each screening stage, these two review authors resolved disagreements through discussion and by involving a third review author (JD) and a clinician expert (EV) if necessary.

We included randomised trials of any designs and non-randomised studies using the designs of non-randomised trials (i.e., experimental trials without random allocations), controlled before-after studies, controlled interrupted time series studies, cohort studies aiming for comparative effectiveness evaluations, and studies with regression discontinuity designs [13, 14]. Eligible non-randomised studies had to evaluate intervention groups comparatively over a defined follow-up time in clearly defined participants and adjust for confounding in the analysis or by study design [14].

We considered studies that compared ‘inpatient-level care at home’ with hospital-based inpatient care as the comparator. We excluded studies that used interventions for care that was not considered acute. Eligible interventions had to use technologies of some form, which could include telephone contact or digital technologies such as apps, wearables. For completeness, we included studies that did not report sufficient information on technology use for us to make a judgement on this criterion, but we did not consider these studies in data analysis. We however explored assumptions around these studies as part of sensitivity analyses (see later section).

### Data extraction

Two authors (CS and FR, AU or SB) independently extracted data for 5% of the included studies to pilot the data extraction form. Then the remaining studies were split into two batches, where one review author extracted data and the other review author checked the data extracted. The pre-prepared data extraction form is publicly accessible at Qualtrics (https://www.qualtrics.manchester.ac.uk/jfe/form/SV_3qQedEEEuYMrhMG) [15]. Our primary outcomes in this review included (1) mortality, measured as proportions of participants who died during study follow-up and (2) number of hospital readmission events following discharge from the episode of care. We considered cost-effectiveness, length of inpatient-level care stay, and adverse events as secondary outcomes.

The form included items of the Template for Intervention Description and Replication (TIDieR) checklist to facilitate full description of models and their components [16]. There is no consensus guidance on the components required for an ‘inpatient-level care at home’ model. We developed our taxonomy using the relevant NHS guidance *Virtual ward including Hospital at Home* [17] and the chronic care model, [18] alongside iterative discussion with health professionals including a virtual ward service lead who contributed substantially to develop and implement virtual ward services. We identified the following five components required to adequately describe innovations in a clinically meaningful way (Additional file 1: Table S1):

- technology involvement (to capture the type of technology being used in the model);
- workforce (to capture who was delivering care);
- clinical activities (to capture what care was being delivered);
- information and support provision (to capture the wider infrastructure and support involved in care delivery); and
- clinical management system used (to capture what types of health record systems were used to support care provision).

Following data extraction, the lead review author (CS) checked and coded components. Then 10% of the included studies were randomly selected, and an independent author (GN) checked the accuracy of the extraction and coding of the models they used.

To synthesise evidence linking the intervention components to effectiveness, we followed the clinically meaningful-element approach to addressing the heterogeneity and complexity of inpatient-level care at home interventions. This approach facilitated us to group those with similar components together, and we were able to further specify clinically important elements using component categories [19, 20]. Whilst we had extracted data on five components, after further discussion and iteration with stakeholders, the main components used to describe the innovations were reduced to three: workforce, clinical activities, and technology involvement only. We considered these three components as the substantive elements needed for inpatient-level care at home to be functional. The two remaining components (information and support provision, and clinical management systems) were considered as secondary features of less direct relevance, and models without these two components may still be functional.

Using a similar approach as with the components themselves, we developed the categories for the three components used across the review to group the models (Table 1). The categories used aimed to capture variation in care models that were relevant to and recognised by those designing and delivering care.

**Table 1:** Categories of components used to define inpatient-level care at home models

Components	Categories	Definitions or comments
(1) Clinical activities	*General inpatient-level care*	General inpatient-level care activities delivered by health professionals based in hospitals, and/or communities, without care activities delivered by allied health professionals. This includes sole activity or treatment delivered such as IV antibiotics at home by hospital outreach nurses
	*Extended multidisciplinary inpatient-level care*	As for general in-patient level care but with additional activities delivered by allied health professionals such as physiotherapists-, occupational, speech therapy, social worker’s support
(2) Workforce	*Hospital ****or**** community-based health professionals*	This category refers to health professionals from either hospital or community settings but not both. This means either community-based clinicians such as general practices, community nurses, and/or communities-based allied health professionals; or hospital-based clinicians including doctors, ward nurses, and/or allied health professionals
	*Hospital ****and**** community-based health professionals*	This category refers to clinicians and/or allied health professionals from both hospitals and communities
(3) Technology involvement	*Low-intensity technology involvement*	This refers to a low-level involvement of technology for a specific purpose. We defined this as use of telephone, and/or teleconferencing for communication alone
	*High-intensity technology involvement*	Use of devices/ technologies such as apps, wearables, digital medical devices, with or without low-intensity-level technologies for multiple purposes at least including communication and remote monitoring. This could include computer stations and fully equipped home-based technology kits that can automatically record health data via a digital platform/ dashboard for communication and continuous monitoring

### Quality assessment of included studies

We used the first version of the Cochrane Risk of Bias tool for randomised controlled trials (RCTs) and the Risk Of Bias In Non-randomised Studies—of Interventions (ROBINS-I) tool for non-randomised studies [21, 22]. In using the ROBINS-I tool for assessing non-randomised studies, we considered age, the severity of primary health conditions, health status, co-morbidities and socioeconomic status as key confounder domains and non-acute care elements as the co-interventions. We acknowledged the importance of considering health inequity issues in this area, and that there were a range of potentially relevant socioeconomic variables. We followed the PROGRESS Plus framework to ensure the thorough consideration of socioeconomic status-related factors reflecting health inequities in this review [23]. The risk of bias assessment involved one author (CS) undertaking the assessment and another author (GN) checking this. All discrepancies were resolved between review authors through discussion.

We referred to the above risk of bias results to appraise the credibility of cost-effectiveness evidence where relevant. We are aware of the reporting checklist Consolidated Health Economic Evaluation Reporting Standards (CHEERS), [24] but we considered it inappropriate to use a reporting checklist for assessing risk of bias of cost-effectiveness analysis. Indeed, developers of the CHEERS checklist clearly advise not to use it as a risk of bias tool [24].

### Data synthesis

We summarised the characteristics of the included studies. Where appropriate, we used meta-analysis with the DerSimonian-Laird random-effects model to combine data across included studies. We assessed heterogeneity from clinical, methodological and statistical perspectives, in which we used the Chi^2^ test and I^2^ statistic to quantify statistical heterogeneity but not to guide effects model choice. We analysed RCTs and non-randomised studies separately.

Rather than performing component-specific analysis, [25, 26] we pooled data by the inpatient-level care at home model groups i.e. those with the same component categories [19]. In this analysis, we took the view that the components of each model have to act in combination to impact on clinical outcomes. We did not then perform a pre-planned sensitivity analysis using component network meta-analysis. We present data using forest plots and present risk ratios (RRs) and mean differences (MDs) with 95% confidence intervals (CIs) for binary and continuous outcomes, respectively.

Whilst our main analyses focus on inpatient-level care at home models grouped by collective component types, we also performed single meta-analyses (one for RCTs and one for non-RCTs) that compared all inpatient-level care at home models with the comparator of hospital-based inpatient care. These post-hoc analyses were undertaken for two purposes: (1) to allow us to broadly assess the consistency between non-randomised and RCT estimates, thus informing appropriateness of using non-randomised evidence as a complement where RCT evidence is unavailable; [27] (2) to reflect the ‘lumping’ approach of previous reviews which has guided decision making to date, in comparison with our more nuanced approach based on component grouping for analyses.

When not undertaking meta-analysis, we considered synthesis of relevant data following the synthesis without meta-analysis in systematic reviews reporting guideline [3, 28]. We used tabular approaches to present available data and report results of narrative synthesis.

We present the main, pooled results of the review in ‘Summary of findings’ tables and assessed the evidence certainty using the Grading Recommendations Assessment and Development Evidence (GRADE) approach [27]. We assessed publication bias using funnel plots and by assessing the comprehensiveness of literature searches [29].

We also performed additional analyses as described in Additional file 1: Text 2 including sensitivity analyses for testing the sensitivity of main analyses to the changes of analysis assumptions.

## Results

### Study selection and characteristics

**Fig. 1:**
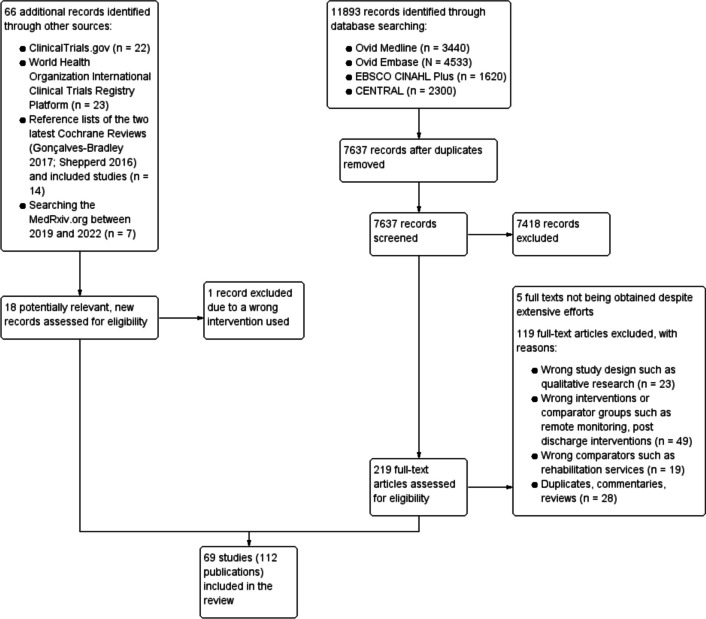
Study selection flowchart

We included a total of 69 studies (Fig. 1) [30–139]: eight (11.6%) were trial registries or protocols for ongoing studies that are still not completed upon writing this report [30, 44–46, 79, 80, 96, 97, 102, 139].

The 69 studies included 38 (55.1%) randomised trials [30–42, 44, 45, 48–50, 54–56, 62–67, 72, 73, 77–83, 92–94, 97, 99, 102–110, 115–122, 125–127, 130–139] and 31 (44.9%) non-randomised studies [43, 46, 47, 51–53, 57–61, 68–71, 74–76, 84–91, 95, 96, 98, 100, 101, 111–114, 123, 124, 128, 129] (Table 2 and Additional file 1: Table S2). The included RCTs were largely judged as overall low (1/38; 2.6%) and unclear risk of bias (25/38; 65.8%; Table 2 and Additional file 1: Table S3). The non-randomised studies were largely at serious (5/31; 16.1%) and critical risk of bias (14/31; 45.2%; Table 2 and Additional file 1: Table S4).

**Table 2:** Summary characteristics of the included studies

Items	Summary statistics across studies (*n* = 69), *n* (%)	Summary statistics for RCTs (*n* = 38), *n* (%)	Summary statistics for non-RCTs (*n* = 31), *n* (%)
***Publication types (n* = *69)***			
Registry record or protocol	8 (11.6%)	6 (15.8%)	2 (6.5%)
Conference abstract	8 (11.6%)	1 (2.6%)	7 (22.6%)
Journal paper	52 (75.4%)	31 (81.6%)	21 (67.7%)
Preprint	1 (1.4%)	0	1 (3.2%)
***Countries (n* = *69)***			
Australia	2 (2.9%)	2 (5.3%)	0
Chile	1 (1.4%)	1 (2.6%)	0
Denmark	3 (4.3%)	3 (7.9%)	0
France	1 (1.4%)	0	1 (3.2%)
Italy	6 (8.7%)	6 (15.8%)	0
Netherlands	2 (2.9%)	2 (5.3%)	0
New Zealand	2 (2.9%)	2 (5.3%)	0
Norway	1 (1.4%)	1 (2.6%)	0
Singapore	1 (1.4%)	0	1 (3.2%)
Spain	8 (11.6%)	2 (5.3%)	6 (19.4%)
Turkey	1 (1.4%)	1 (2.6%)	0
UK	22 (31.9%)	13 (34.2%)	9 (29%)
Uruguay	1 (1.4%)	0	1 (3.2%)
US	15 (21.7%)	5 (13.1%)	10 (32.3%)
Unspecified	3 (4.3%)	0	3 (9.7%)
***Primary conditions (n* = *69)***			
General unspecified acute medical conditions	24 (34.8%)	11 (28.9%)	13 (41.9%)
Acute decompensation of chronic heart failure	3 (4.3%)	3 (7.9%)	0
Acute respiratory conditions such as pneumonia, chronic obstructive pulmonary disease (COPD) acute exacerbations, neuromuscular disease patients with severe respiratory tract infection	17 (24.6%)	11 (28.9%)	6 (19.4%)
Cystic fibrosis or pulmonary exacerbations	3 (4.3%)	0	3 (9.7%)
Specific acute conditions such as acute calculous cholecystitis, acute pancreatitis, acute uncomplicated diverticulitis, or exacerbations of chronic conditions such as stroke, cancer patients who need acute care	10 (14.5%)	6 (15.8%)	4 (12.9%)
Surgical patients such as hip and knee replacement, fractured neck of femur, inguinal hernia or varicose veins, coronary artery bypass grafting	5 (7.2%)	2 (5.3%)	3 (9.7%)
Hospitalised moderately ill children, preterm infants	2 (2.9%)	1 (2.6%)	1 (3.2%)
Older people including those of frailty or prior dependence and those with cognitive impairment who are in need of acute hospital care	4 (5.8%)	4 (10.5%)	0
Unspecified	1 (1.4%)	0	1 (3.2%)
***Sample sizes (n* = *60)***			
Summaries	Total = 38,363 participants; Median 115.5 (range 21 to 22,610)	Total = 6413 participants in 32 studies; Median 111, (range 21 to 1055)	31,950 in 28 studies; Median 142 (range 30 to 22,610)
The number of participants in interventions	10,229 participants	2817 participants in 29 studies	7412 in 29 studies
The number of participants in control	27,824 participants	2501 participants in 29 studies	25,323 in 28 studies
***Average age in years (n* = *44)***	Median 70.3 (range 2.1 to 83.3)	Median 70 in 22 studies (range 2.1 to 83.3)	Median 74 in 22 studies (range 12.6 to 81.7)
***Sex (n* = *46)***			
Male	15,891 participants	2256 in 27 studies	13,635 in 19 studies
Female	19,275 participants	2804 in 27 studies	16,471 in 19 studies
***Inpatients level care at home interventions***	63 interventions	33 interventions	30 interventions
Unspecified	12 (19.0%)	4 (12.1%)	8 (26.7%)
Early discharge	22 (34.9%)	15 (45.5%)	7 (23.3%)
Avoidance of hospital admission	25 (39.7%)	13 (39.4%)	12 (40%)
Avoidance of hospital admission and early discharge	4 (6.3%)	1 (3.0%)	3 (10%)
***Intervention groups***	63 interventions	33 interventions	30 interventions
General inpatient-level care, delivered by hospital- or community-based professionals, with high-intensity technology involvement	7 (11.1%)	3 (9.1%)	4 (13.3%)
General inpatient-level care, delivered by hospital- or community-based professionals, with low-intensity technology involvement	16 (25.4%)	11 (33.3%)	5 (16.6%)
General inpatient-level care, delivered by hospital- and community-based professionals, with high-intensity technology involvement	2 (3.2%)	1 (3.0%)	1 (3.3%)
General inpatient-level care, delivered by hospital- and community-based professionals, with low-intensity technology involvement	1 (1.6%)	1 (3.0%)	0
Extended multidisciplinary inpatient-level care, delivered by hospital- or community-based professionals, with high-intensity technology involvement	2 (3.2%)	2 (6.1%)	0
Extended multidisciplinary inpatient-level care, delivered by hospital- or community-based professionals, with low-intensity technology involvement	4 (6.3%)	2 (6.1%)	2 (6.7%)
Extended multidisciplinary inpatient-level care, delivered by hospital- and community-based professionals, with low-intensity technology involvement	9 (14.3%)	5 (15.2%)	4 (13.3%)
Interventions that have no detail of technology, workforce, or care pathway, or the three components	22 (34.9%)	8 (24.2%)	14 (46.7%)
***Follow up (n* = *49)***	Median 3 months (range 7 days to 12 months)	Across 32 studies: Median 3 months (range 7 days to 12 months)	Across 17 studies: Median 3 months (range 1 month to 12 months)
***Overall risk of bias (n* = *69)***			
Cochrane risk of bias			
Low		1 (2.6%)	NA
Unclear		25 (65.8%)	NA
High		4 (10.5%)	NA
Not applicable for trial registries or protocols		8 (21.1%)	
ROBINS-I			
Low		NA	0
Moderate		NA	3 (9.7%)
Serious		NA	5 (16.1%)
Critical		NA	14 (45.2%)
No information		NA	9 (29%)

The 69 studies described 63 unique inpatient-level care at home interventions (Additional file 1: Table S57), of which 25 (39.7%) were reported as aiming to avoid hospital admissions, 22 (34.9%) were for early discharge, 4 (6.3%) were for both purposes, and 12 (19.0%) had no relevant detail. Based on the three components of focus, the 63 interventions formed eight model groups for analyses (Table 2).

### Effects of inpatient-level care at home

We present evidence separately for RCTs and non-randomised studies for mortality, hospital readmission and length of stay (Additional file 1: Table S6; Additional file 2: Fig. S1 to S6). Non-randomised data clearly overestimated the effectiveness in our post hoc exploratory analyses compared with randomised data (Additional file 1: Text 2), and we summarise analyses of RCT data below and only present non-randomised data where RCT evidence is unavailable (Table 3). As random-effects models were used, all findings are average effects.

**Table 3:** High-level summaries of findings for data analyses by model groups

Clinical activities	Workforce	Technology involvement	Mortality	Hospital readmission	Length of care stay in days
General inpatient-level care	Hospital or community-based professionals	Low intensity	Uncertain evidence (RR 0.58, 95% CI 0.20 to 1.71; low-certainty RCT evidence)	There may be no difference between technology-enabled models of this group and hospital-based inpatient care, but there are some uncertainties around this as the confidence interval includes risk of benefits and harms (RR 1.03, 95% CI 0.79 to 1.34; low-certainty RCT evidence)	There may be no difference between technology-enabled models of this group and hospital-based inpatient care, but there are some uncertainties around this as the confidence interval includes risk of benefits and harms (MD 0.29, 95% CI − 2.56 to 3.14; low-certainty RCT evidence)
		High intensity	Uncertain evidence (RR 0.72, 95% CI 0.18 to 2.95; low-certainty RCT evidence)	Uncertain evidence (RR 0.65, 95% CI 0.22 to 1.93; very low-certainty non-RCT evidence)	Uncertain evidence (MD − 1.07, 95% CI − 2.04 to − 0.10; very low-certainty non-RCT evidence)
	Hospital- and community-based professionals	Low intensity	Technology-enabled models of this group may have a lower mortality incidence on average than hospital-based inpatient care (RR 0.29, 95% CI 0.09 to 0.95; low-certainty RCT evidence)	Technology-enabled models of this group may have a lower readmission incidence on average than hospital-based inpatient care (RR 0.65, 95% CI 0.40 to 1.06; low-certainty RCT evidence)	No evidence available
		High intensity	Uncertain evidence (RR 0.78, 95% CI 0.19 to 3.15; low-certainty RCT evidence)	Technology-enabled models of this group may have a lower readmission incidence on average than hospital-based inpatient care (RR 0.37, 95% CI 0.23 to 0.60; low-certainty non-RCT evidence)	No evidence available
Extended multidisciplinary inpatient-level care	Hospital or community-based professionals	Low intensity	Uncertain evidence (RR 0.97, 95% CI 0.06 to 15.09; low-certainty RCT evidence)	There may be little to no difference between technology-enabled models of this group and hospital-based inpatient care, but there are some uncertainties around this as the confidence interval includes risk of benefits and harms (RR 0.92, 95% CI 0.58 to 1.46; low-certainty RCT evidence)	Technology-enabled models of this group may have a shorter stay on average than hospital-based inpatient care (MD − 2.9, 95% CI − 4.2 to − 1.6; low-certainty RCT evidence)
		High intensity	No evidence available	Technology-enabled models of this group may have a lower readmission incidence on average than hospital-based inpatient care (RR 0.30, 95% CI 0.11 to 0.86; low-certainty RCT evidence)	There may be no difference between technology-enabled models of this group and hospital-based inpatient care (MD 0.46, 95% CI − 0.22 to 1.14, low-certainty RCT evidence)
	Hospital- and community-based professionals	Low intensity	There is probably no difference between technology-enabled models of this group and hospital-based inpatient care (RR 0.96, 95% CI 0.79 to 1.16; moderate-certainty RCT evidence)	There may be little to no difference between technology-enabled models of this group and hospital-based inpatient care, but there are some uncertainties around this as the confidence interval includes risk of benefits and harms (RR 0.94, 95% CI 0.69 to 1.28; low-certainty RCT evidence)	Technology-enabled models of this group may have longer stay on average than hospital-based inpatient care (4.85, 95% CI 1.8 to 7.9 days; low-certainty RCT evidence)
		High intensity	No evidence available	No evidence available	No evidence available

#### Mortality

RCT evidence is uncertain or unavailable for six models (Table 3 and Additional file 1: Table S6; Additional file 2: Fig. S1 and Fig. S2). There is low- or moderate-certainty evidence of, on average, similar or lower mortality risk than hospital-based inpatient care for two models: general inpatient-level care (RR 0.29, 95% CI 0.09 to 0.95; low-certainty RCT evidence) and extended multidisciplinary inpatient-level care (RR 0.96, 95% CI 0.79 to 1.16; moderate-certainty RCT evidence), delivered by hospital- and community-based professionals, with low-intensity technology involvement.

#### Hospital readmission

Six at home models have low-certainty evidence available, all suggesting, on average, at least a similar or lower risk of hospital readmission in people allocated to the inpatient-level care at home arms. Two models have uncertain or unavailable evidence (Table 3 and Additional file 1: Table S6; Additional file 2: Fig. S3 and Fig. S4).

#### Cost-effectiveness

Only two UK studies reported cost‐effectiveness and/or cost-utility analyses (Additional file 1: Text 3), both based on well-conducted trials but reporting conflicting evidence [65, 66]. A small trial-based analysis (118 participants, with 90-day follow-up) suggested that the model evaluated (i.e. extended multidisciplinary inpatient-level care, delivered by hospital- or community-based professionals, with low technology involvement) may be less expensive but more effective than hospital-based inpatient care for people with COPD exacerbations. The reported probability of this model being cost-effective was 90% at the National Institute for Health and Care Excellence’s threshold of £30,000 per quality-adjusted life year (QALY) gained [65]. The other analysis, based on a larger trial (457 stroke participants with 12-month follow-up), did not specify the technology its model used but suggested that its probability of being cost-effective is only 42% at the above threshold [66].

#### Length of stay

People receiving an at home model composed of extended multidisciplinary inpatient-level care, delivered by hospital- and community-based professionals, with low-intensity technology involvement may have, on average, a length of stay 4.85 days (95% CI 1.8 to 7.9 days) longer than hospital-based inpatient care (low-certainty RCT evidence). Three of the other models had, on average, similar or shorter length of care stays compared with hospital-based inpatient care. Four models have unavailable or uncertain evidence (Table 3 and Additional file 1: Table S6; Additional file 2: Fig. S5 and Fig. S6).

#### Adverse events

Four RCTs and seven non-randomised studies reported this outcome [31–33, 36–38, 52, 53, 60, 68, 74, 81–84, 87–91, 101]. We performed no synthesis for this outcome as the outcomes used were defined inconsistently, and outcome data were incomplete in four studies. The data, where available, appear to suggest conflicting evidence on the adverse effects of using hospital-level care at home (Additional file 1: Table S7).

### Additional analyses

Pre-specified and post hoc sensitivity analyses suggest that the main analyses were not sensitive to analysis assumptions used (Additional file 1: Text 2), including: assuming that low technologies were used for interventions that had no information on technology use in the related studies (*n* = 22).

Our post hoc explanatory analyses that grouped all interventions into a broader comparison between inpatient-level care at home and hospital-based inpatient care suggested no statistical difference in mortality and readmission risk between groups. There appears to be an increase in the length of stay in inpatient-level inpatient care (Additional file 1: Text 2). Contrasting these findings with the model group-specific analyses above highlights the value of linking components to effectiveness.

## Discussion

### Summary of findings

Use of technology-enabled inpatient-level care at home (including models with low-intensity technology such as phone contact) may result in a similar risk of readmission to hospital following discharge compared with those receiving their initial care in hospital. The evidence is largely unavailable or uncertain for mortality and is mixed for length of stay. Where there is evidence on mortality, there may be no additional risk of mortality due to use of technology-enabled at home models. Cost-effectiveness evidence is unavailable for high technology-enabled models, and there is only limited evidence suggesting that the low technology-enabled multidisciplinary care model delivered by hospital-based teams may be cost-effective for people with COPD exacerbations.

### Evidence in context

The current pressures in urgent and emergency services are promoting the expansion of technology-enabled, innovative care at home models, including virtual wards. Over the medium to longer term, expanding such innovations represents a paradigm shift in how acute care is delivered so that hospital occupancy can be better managed [140]. Care at home models are expected to become more integrated parts of future healthcare system with the continuing development of relevant technologies such as telehealth platforms, wearables, predictive algorithms including artificial intelligence. NICE health technology evaluation guidance (HTE13) has summarised key features that future virtual ward platform technologies should have, including interoperability with electronic record systems and medical devices; risk-stratified alerts; trend-based alerts; and patient interface with a user-centred design [141].

Our novel review highlights the value of disaggregating inpatient-level care at home models into constituent components to allow a more nuanced presentation of existing research findings. Our detailed analyses by component permutations provide a framework for future evaluations. The rapid scale-up of virtual wards in the UK and internationally includes a strong emphasis on high-intensity technology involvement [17, 142]. Limited available evidence means we are unclear whether use of high-intensity technology in these models confers additional benefit compared with hospital-based inpatient care. Further implementation of these models will benefit from concomitant evaluation with a focus on the added value of more complex technologies. Where the availability of high-intensity technology is a barrier to the implementation and evaluation of inpatient-level care at home models, lower-intensity models can be considered, again with evaluation.

For the key component of workforce, this review suggests that inpatient-level care at home delivered by hospital- and community-based professionals could result in similar readmission and/or mortality incidence to hospital-based inpatient care. This suggests the importance of coordination between hospital- and community-based teams to ensure the continuity of inpatient care in clinical practice [143]. The impacts of the team coordination on primary care workforce and provision require evaluations but are seldom reported. Existing evaluations focus on inpatient care provision and outcomes as opposed to relevant issues in primary care settings.

When considering the relevance of the evidence base in the UK, respiratory conditions, heart failure, and frailty are high-priority populations for inpatient-level care at home [17]. Whilst almost a quarter of the studies we included focused on acute respiratory conditions, our review highlights the limited available evidence for populations with frailty (four RCTs, with 1735 participants), and heart failure (three RCTs with 224 participants). The development of inpatient-level care at home models for frail and other high-priority populations should be informed by relevant existing evidence whilst recognising the likely need for carefully planned, likely rapid, evaluations.

### Implications for research

In future, rigorous evaluation research is required to support the on-going development and implementation of technology-enabled inpatient-level care at home models and guide future decision-making about the value gained for investment. RCTs would be the ideal study design, but there are challenges to this. Non-randomised studies are more feasible, and these designs should evaluate intervention groups comparatively over a clearly defined, sufficient follow-up time in well-defined participants and appropriately adjust for confounding [14]. There is a crucial role for routinely collected data to allow rapid evaluation of this service delivery model, and identifying flags for service use should be added to data systems as far as possible. Intervention design and the corresponding evaluation should map to the approach of this review or other work that has considered the key elements of technology-enabled models [143, 144]. Important outcomes are not limited to those used in this review but also include the experiences of patient and caregivers.

### Comparisons with other studies

In a rapid evidence synthesis, our scoping search identified 11 published systematic reviews of interventions that were described as ‘hospital at home’ or ‘virtual wards’ in comparison with hospital-based inpatient care, in a range of populations [6]. We found that there is low- to moderate-certainty evidence, suggesting that the interventions described as ‘hospital at home’ are probably as good or better than hospital-based inpatient care in terms of clinical outcomes including mortality and readmission [6]. In the previous reviews, the evidence is inconsistent on virtual wards for readmission to hospital [6].

In comparison with other work, our review focused on technology-enabled inpatient-level care at home models for people with acute conditions who would otherwise require hospitalisation. Previous reviews identified in this area included not only inpatient-level care at home but also remote monitoring that may not involve inpatient-level care [6]. This is an important distinction and impacts on the generalisability of review findings to specific intervention types. Unlike previous reviews, we present evidence for eight permutations of three components (Table 3), allowing stakeholders to refer to relevant evidence based on characteristics of the models used in their practice.

In previous reviews, the evidence for ‘virtual wards’ has appeared inconsistent regarding the effectiveness in reducing hospital (re)admission, depending on care models and health conditions [6]. We found consistency in this outcome across inpatient-level care at home models, and the available evidence suggests the interventions may have at least an equivalent readmission incidence to hospital-based inpatient care.

## Strengths and limitations of this review

This review has strengths partly due to use of standard Cochrane-based, prespecified review methods in minimising the risk of bias in the review process. Use of a comprehensive search identified far more studies than existing systematic reviews on this topic [6]. In defining eligibility criteria, we considered use of digital technologies and the substitution of hospital-based inpatient care in the home environment as two important elements for inpatient-level care at home [17]. This ensures that the evidence is in line with the current innovations [17]. Given that there is no consensus on ‘inpatient-level care at home’ components, we developed and defined a typology. This helps ensure that components chosen are clinically relevant and the process is trustworthy. We noted that the components chosen via our approach are reflected in the recent relevant reviews that identified key elements for building virtual wards [143, 144].

This review has limitations. These include the widely acknowledged challenge in identifying non-randomised studies for inclusion in a review, [13] and the lack of an agreed single approach to grouping interventions. For these issues, we followed the Cochrane Effective Practice and Organisation of Care (EPOC) guidance and Reeves et al.’s checklist to minimise study selection bias [13, 14]. Given inpatient-level care at home is a heterogeneous set of complex interventions whose theories of change are not well defined, we followed the clinically meaningful-element approach to grouping inpatient-level care at home interventions for informing subsequent synthesis [19]. That is, we grouped the interventions with similar components together whilst considering clinically important elements in specifying component categories [19]. This approach allows us to investigate which combinations of components are associated with intervention effectiveness [20]. We reached a consensus on the clinically sensible grouping in consultation with methodological and clinical experts including a clinical virtual ward lead. Further research is needed to build on the first attempt in defining intervention components presented in this review, and to develop and validate intervention taxonomies in this area. We were unable to determine which model components are important, as component-specific analysis was considered inappropriate in this review (as justified in the ‘Methods’ section). In coding components of the included interventions, the lead review author followed the agreed process, with a second review author checking 10% of the included studies for comparison. We noted that the decision of using 10% of the included studies for comparison was arbitrary but pragmatic. Whilst this approach may have increased the risk of coding errors, empirically, this risk was limited here because the agreement in the 10% of randomly sampled papers was very good. We suggest this was due to the careful development and piloting of the process and the experience of the reviewers involved. We did not assess the quality of cost-effectiveness evaluations included, [24] but we noted the relevant trials used in these evaluations were small with short follow-ups and had no substantial methodological limitations.

## Conclusions

We found that a range of technology-enabled inpatient-level care at home models may result in similar or reduced readmission risk compared with hospital-based inpatient care. Impacts on mortality are more uncertain, except for two models showing no increased risk compared with hospital-based inpatient care. The certainty of current evidence means further research could change findings. Further implementation of inpatient-level care at home models should be alongside evaluation to explore the potential benefits of using specific technologies particularly to gain further insights into clinical and cost-effectiveness particularly in high-priority populations.

### Supplementary Information

**Additional file 1: Text 1.** Literature search methods used. **Table S1.** Intervention components of inpatient level care at home and specifications. **Text 2.** Methods and results of subgroup and sensitivity analyses. **Table S2.** Characteristics of the included studies. **Table S3.** Risk of bias assessment results for RCTs. **Table S4.** Risk of bias assessment results for non-randomised studies. **Table S5.** Summarises of intervention components. **Table S6.** Summary of findings tables for individual outcomes. **Text 3.** Results of cost and cost-effectiveness analyses. **Table S7.** Results of adverse event outcomes.

**Additional file 2: Fig. S1.** Meta-analyses of RCT data by care models for mortality. **Fig. S2.** Meta-analyses of non-randomised data by care models for mortality. **Fig. S3.** Meta-analyses of RCT data by care models for hospital readmission. **Fig. S4.** Meta-analyses of non-randomised data by care models for hospital readmission. **Fig. S5.** Meta-analyses of RCT data by care models for the length of care stay in days. **Fig. S6.** Meta-analyses of non-randomised data by care models for the length of care stay in days.

## Data Availability

Data extracted from included studies and data used for analyses are all reported in this paper or its appendices. No additional data are available.

## Abbreviations

CHEERS: Consolidated Health Economic Evaluation Reporting Standards
CI: Confidence interval
COPD: Chronic obstructive pulmonary disease
EPOC: Cochrane Effective Practice and Organisation of Care
GRADE: Grading Recommendations Assessment and Development Evidence
MD: Mean difference
NHS: National Health Service
QALY: Quality-adjusted life year
RCT: Randomised controlled trial
ROBINS-I: Risk Of Bias In Non-randomised Studies—of Interventions
RR: Risk ratio
TIDieR: Template for Intervention Description and Replication
