# Supplementary material for: Inpatient-level care at home delivered by virtual wards and hospital at home: a systematic review and meta-analysis of complex interventions and their components
Source: BMC Med. 2024 Apr 2;22:145. doi: 10.1186/s12916-024-03312-3 (PMC10986022; doi:10.1186/s12916-024-03312-3)
Supplement: Supplementary file 1 — Additional file 1: Text 1. Literature search methods used. Table S1. Intervention components of inpatient level care at home and specifications. Text 2. Methods and results of subgroup and sensitivity analyses. Table S2. Characteristics of the included studies. Table S3. Risk of bias assessment results for RCTs. Table S4. Risk of bias assessment results for non-randomised studies. Table S5. Summarises of intervention components. Table S6. Summary of findings tables for individual outcomes. Text 3. Results of cost and cost-effectiveness analyses. Table S7. Results of adverse event outcomes. [file 12916_2024_3312_MOESM1_ESM.docx]

**Text 1. Literature search methods used**

In developing the search strategies, we were advised by a Cochrane information specialist (Sophie Bishop) and referred to (1) ‘virtual ward’ and ‘hospital at home’ search terms used in existing Cochrane Reviews, the Cochrane EPOC study design filters for non-randomised studies and other relevant filters. We limited searches to English language publications due to limited resources. There was no restriction with respect to date of publication or study setting.

Below is the search strategy used for searching Ovid MEDLINE(R) and Epub Ahead of Print, In-Process, In-Data-Review & Other Non-Indexed Citations, Daily and Versions <1946 to November 29, 2022>

1 randomized controlled trial.pt. 581696

2 controlled clinical trial.pt. 95114

3 (randomis* or randomiz* or randomly).ti,ab. 1078961

4 multicenter study.pt. 328012

5 pragmatic clinical trial.pt. 2168

6 (trial or multicenter or multi center or multicentre or multi centre).ti. 331289

7 comparative study/ 1911803

8 prospective studies/ 644781

9 follow-up studies/ 688605

10 exp Cohort studies/ 2421078

11 exp clinical trial/ 956794

12 exp evaluation studies as topic/ 1190082

13 (quasiexperiment* or quasi experiment* or pseudo experiment* or pseudoexperiment*).ti,ab. 19635

14 interrupted time series.ti,ab. 5074

15 (controlled adj3 before adj3 after).ti,ab. 1632

16 regression discontinuity.ti,ab. 576

17 cohort.mp. 848194

18 non-randomized controlled trials as topic/ 1049

19 interrupted time series analysis/ 1725

20 controlled before-after studies/ 707

21 controlled.ti,ab. 921247

22 control group?.ti,ab. 544891

23 (before adj5 after).ti,ab. 434963

24 (pre adj5 post).ti,ab. 122776

25 ((pretest or pre test) and (posttest or post test)).ti,ab. 15249

26 time series.ti,ab. 41622

27 compared.mp. 4198848

28 groups.mp. 2547237

29 multivariate.mp. 480436

30 exp animals/ 25976106

31 humans/ 20906265

32 30 not (30 and 31) 5069841

33 review.pt. 3080992

34 meta analysis.pt. 171626

35 news.pt. 215616

36 comment.pt. 987596

37 editorial.pt. 628035

38 cochrane database of systematic reviews.jn. 16074

39 comment on.cm. 987543

40 (systematic review or literature review).ti. 250307

41 32 or 33 or 34 or 35 or 36 or 37 or 38 or 39 or 40 9692261

42 1 or 2 or 3 or 4 or 5 or 6 or 7 or 8 or 9 or 10 or 11 or 12 or 13 or 14 or 15 or 16 or 17 or 18 or 19 or 20 or 21 or 22 or 23 or 24 or 25 or 26 or 27 or 28 or 29 10589606

43 (virtual adj3 ward*).ti,ab. 88

44 (virtual adj3 monitor*).ti,ab. 167

45 (home* adj3 monitor*).ti,ab. 6265

46 hospital at home.ti,ab. 569

47 43 or 44 or 45 or 46 7058

48 42 and 47 3910

49 48 not 41 3440

**Table S1. Intervention components of inpatient level care at home and specifications**

| **Components** | **Component outline** | **Description** | **Purpose** | **Mechanism** |
| --- | --- | --- | --- | --- |
| ***Digital technology*** | Digital technology is the defining feature for technology-enabled inpatient level care at home models. The technologies involved can be at a low level such as telephone, or at a higher level such as designated computer-based monitoring station, wearable devices, apps. The technologies can be used for remote monitoring, vital sign measuring and communications. Digital devices can be provided to patients as part of the hospital care at home programme. | What (type of technology):   - Low tier such as telephone - Apps - Wearables - Internet - Digital medical devices - Computer station | For what (purpose of use)   - Communication - Monitoring - Measuring |  |
| **Workforce** | Workforce may vary between specific models. Hospital care at home models should at least involve hospital-based teams for e.g. remote consultations, and central monitoring, but could involve hospital outreach team or even community-based teams for e.g. face-to-face in-person care delivery. The team is often multidisciplinary. | What (workforce type)   - Consultant - Hospital outreach team - Community team - Non-health professionals such as hospital-based remote monitoring team, administration clerk | For what (responsibility of workforce)   - Face to face in-person care delivery - Remote consultation - Remote monitoring - Management/ administration |  |
| **Care planning, management and provision** | Similar with hospital-based care, a consultant practitioner will daily review the care of patients admitted to a hospital care at home daily. This can involve office-based ‘ward rounds’ supported by a digital platform, in which a patient can be escalated to a multidisciplinary care. If required, a patient can have access to specialty treatment and diagnostics equivalent to acute hospital care. | What (types of care planning, management and provision)   - Ward rounds - Triage - Admission - Diagnostics - (Specialty) treatment - Discharge - Advanced multidisciplinary care |  | How   - Technology-enabled - In-person   How long   - Within 14 days - Longer than 14 days   How often (to be specified) |
| **Information and support provision** | Hospital care at home often need the self-management of patients or support of family caregivers. This model should provide educational materials or information, equivalent to acute hospital care, that can allow patients and/or their family caregivers to understand their care, and if required, the use of digital technology or devices such as wearables, apps. | What (types of provision)   - Information support - Provision of devices such as a designated pack of devices - Consultation support - Technology use support   To whom   - Primary care or outreach practitioner, or peer - Patients - Family caregivers | For what   - Delivering care - Educating patients - Peer support | How often (to be specified) |
| ***Clinical management system*** | This model can involve the use of digital patient records to ensure access to clinical information at the point of care to support provision of care. | What (type of system)   - Electronic record system - Interoperable care record system - Paper based approaches |  |  |

**Text 2. Methods and results of subgroup and sensitivity analyses***

**Subgroup analysis.** We did not perform the pre-planned subgroup analysis for exploring subgroup effects for different health condition categories such as acute respiratory conditions, older people including frailty. This is because none of the main analyses by model groups included more than 10 studies.

**Sensitivity analysis**. We assessed the robustness of meta-analysis by testing their sensitivity to: changing the effects model used; and excluding studies at overall high risk of bias. We did not perform a planned sensitivity analysis around missing data because information reporting in included studies did not allow this.

To assess the impact of our approach to model grouping we performed post hoc sensitivity analyses: (1) merging general inpatient-level care and extended multidisciplinary inpatient-level care categories for the clinical activity component; (2) assuming the 22 studies excluded from the main analyses due to no reported technology involvement used low intensity technology; (3) grouping all models into four higher-level categories based on their care approaches of: admission avoidance; early discharge; admission avoidance and early discharge; and unspecified models.

Results of these pre-planned and post hoc sensitivity analyses are presented in the table below.

| **Grouping using the following three components** | | | **Mortality** | **Hospital readmission** | **Length of stay in days** |
| --- | --- | --- | --- | --- | --- |
| **Clinical activities** | **Workforce** | **Technology involvement** |  |  |  |
| General inpatient care | Hospital or community-based professionals | Low intensity | Main analysis. RR 0.58, 95% CI 0.20 to 1.71  Sensitivity analysis 1. RR 0.58, 95% CI 0.20 to 1.71  Sensitivity analysis 2. RR 0.57, 95% CI 0.20 to 1.65  Sensitivity analysis 3. RR 0.69, 95% CI 0.20 to 2.40 | Main analysis. RR 1.03, 95% CI 0.79 to 1.34  Sensitivity analysis 1. RR 1.03, 95% CI 0.79 to 1.34  Sensitivity analysis 2. RR 1.09, 95% CI 0.83 to 1.42  Sensitivity analysis 3. RR 1.09, 95% CI 0.82 to 1.45 | Main analysis. MD 0.29, 95% CI -2.56 to 3.14  Sensitivity analysis 1. MD -0.48, 95% CI -3.02 to 2.06  Sensitivity analysis 2. MD 0.85, 95% CI 0.55 to 1.15  Sensitivity analysis 3. MD 0.29, 95% CI -2.56 to 3.14 |
|  |  | High intensity | Main analysis. RR 0.72, 95% CI 0.18 to 2.95  Sensitivity analysis 1. RR 0.72, 95% CI 0.18 to 2.95  Sensitivity analysis 2. RR 0.72, 95% CI 0.18 to 2.95  Sensitivity analysis 3. RR 0.72, 95% CI 0.18 to 2.95 | Main analysis. RR 0.65, 95% CI 0.22 to 1.93 (non-RCT)  Sensitivity analysis 1. RR 0.65, 95% CI 0.22 to 1.93 (non-RCT)  Sensitivity analysis 2. RR 0.89, 95% CI 0.65 to 1.22 (non-RCT)  Sensitivity analysis 3. No data available | Main analysis. MD -1.07, 95% CI -2.04 to -0.10 (non-RCT)  Sensitivity analysis 1. MD -1.07, 95% CI -2.04 to -0.10 (non-RCT)  Sensitivity analysis 2. MD -1.25, 95% CI -1.54 to -0.96 (non-RCT)  Sensitivity analysis 3. No data available |
|  | Hospital- and community-based professionals | Low intensity | Main analysis. RR 0.29, 95% CI 0.09 to 0.95  Sensitivity analysis 1. RR 0.58, 95% CI 0.15 to 2.18  Sensitivity analysis 2. RR 0.29, 95% CI 0.09 to 0.95  Sensitivity analysis 3. RR 0.29, 95% CI 0.09 to 0.95 | Main analysis. RR 0.65, 95% CI 0.40 to 1.06  Sensitivity analysis 1. RR 0.85, 95% CI 0.51 to 1.40  Sensitivity analysis 2. RR 0.65, 95% CI 0.40 to 1.06  Sensitivity analysis 3. RR 0.65, 95% CI 0.40 to 1.06 | Unavailable |
|  |  | High intensity | Main analysis. RR 0.78, 95% CI 0.19 to 3.15  Sensitivity analysis 1. RR 0.78, 95% CI 0.19 to 3.15  Sensitivity analysis 2. RR 0.78, 95% CI 0.19 to 3.15  Sensitivity analysis 3. RR 0.78, 95% CI 0.19 to 3.15 | Main analysis. RR 0.37, 95% CI 0.23 to 0.60 (non-RCT)  Sensitivity analysis 1. RR 0.37, 95% CI 0.23 to 0.60 (non-RCT)  Sensitivity analysis 2. RR 0.37, 95% CI 0.23 to 0.60 (non-RCT)  Sensitivity analysis 3. RR 0.37, 95% CI 0.23 to 0.60 (non-RCT) | Unavailable |
| Extended multidisciplinary services | Hospital or community-based professionals | Low intensity | Main analysis. RR 0.97, 95% CI 0.06 to 15.09  Sensitivity analysis 1. RR 0.97, 95% CI 0.06 to 15.09  Sensitivity analysis 2. RR 0.97, 95% CI 0.06 to 15.09  Sensitivity analysis 3. RR 0.97, 95% CI 0.06 to 15.09 | Main analysis. RR 0.92, 95% CI 0.58 to 1.46  Sensitivity analysis 1. RR 0.92, 95% CI 0.58 to 1.46  Sensitivity analysis 2. RR 0.92, 95% CI 0.58 to 1.46  Sensitivity analysis 3. RR 0.92, 95% CI 0.58 to 1.46 | Main analysis. MD -2.90, 95% CI -4.2 to -1.6  Sensitivity analysis 1. MD -2.90, 95% CI -4.2 to -1.6  Sensitivity analysis 2. MD -2.90, 95% CI -4.20 to -1.60  Sensitivity analysis 3. MD -2.90, 95% CI -4.20 to -1.60 |
|  |  | High intensity | Unavailable | Main analysis. RR 0.30, 95% CI 0.11 to 0.86  Sensitivity analysis 1. RR 0.30, 95% CI 0.11 to 0.86  Sensitivity analysis 2. RR 0.30, 95% CI 0.11 to 0.86  Sensitivity analysis 3. RR 0.30, 95% CI 0.11 to 0.86 | Main analysis. MD 0.46, 95% CI -0.22 to 1.14  Sensitivity analysis 1. MD 0.46, 95% CI -0.22 to 1.14  Sensitivity analysis 2. MD 0.46, 95% CI -0.22 to 1.14  Sensitivity analysis 3. MD 0.46, 95% CI -0.22 to 1.14 |
|  | Hospital- and community-based professionals | Low intensity | Main analysis. RR 0.96, 95% CI 0.79 to 1.16  Sensitivity analysis 1. RR 0.95, 95% CI 0.80 to 1.13  Sensitivity analysis 2. RR 0.97, 95% CI 0.80 to 1.18  Sensitivity analysis 3. RR 0.94, 95% CI 0.78 to 1.15 | Main analysis. RR 0.94, 95% CI 0.69 to 1.28  Sensitivity analysis 1. RR 0.93, 95% CI 0.69 to 1.24  Sensitivity analysis 2. RR 1.00, 95% CI 0.89 to 1.12  Sensitivity analysis 3. RR 0.87, 95% CI 0.60 to 1.25 | Main analysis. 4.85, 95% CI 1.8 to 7.9  Sensitivity analysis 1. MD 3.88, 95% CI 2.11 to 5.66  Sensitivity analysis 2. MD 2.58, 95% CI 1.86 to 3.29  Sensitivity analysis 3. MD 5.85, 95% CI 0.61 to 11.09 |
|  |  | High intensity | Unavailable | Unavailable | Unavailable |
|  | | | | | |
| **Grouping using workforce and technology involvement components only (sensitivity analysis 4)** | | | **Mortality** | **Hospital readmission** | **Length of stay in days** |
|  | Hospital or community-based professionals | Low intensity | Sensitivity analysis 4. RR 0.62, 95% CI 0.23 to 1.70 | Sensitivity analysis 4. RR 1.00, 95% CI 0.80 to 1.26 | Sensitivity analysis 4. MD -0.51, 95% CI -3.09 to 2.07 |
|  |  | High intensity | Sensitivity analysis 4. RR 0.72, 95% CI 0.18 to 2.95 | Sensitivity analysis 4. RR 0.30, 95% CI 0.11 to 0.86 | Sensitivity analysis 4. MD 0.46, 95% CI -0.22 to 1.14 |
|  | Hospital and community-based professionals | Low intensity | Sensitivity analysis 4. RR 0.93, 95% CI 0.75 to 1.13 | Sensitivity analysis 4. RR 0.90, 95% CI 0.68 to 1.19 | Sensitivity analysis 4. MD 4.85, 95% CI 1.80 to 7.91 |
|  |  | High intensity | Sensitivity analysis 4. RR 0.78, 95% CI 0.19 to 3.15 | Sensitivity analysis 4. RR 0.37, 95% CI 0.23 to 0.60 (non-RCT) | Sensitivity analysis 4. No data available for analysis |
|  | | | | | |
| **Groups of admission avoidance, early discharge or both** | | | **Mortality** | **Hospital readmission** | **Length of stay in days** |
|  | | Admission avoidance | Sensitivity analysis 5. RR 0.94, 95% CI 0.79 to 1.12 | Sensitivity analysis 5. RR 0.89, 95% CI 0.68 to 1.17 | Sensitivity analysis 5. MD 2.80, 95% CI 1.32 to 4.27 |
|  |  | Early discharge | Sensitivity analysis 5. RR 0.63, 95% CI 0.35 to 1.12 | Sensitivity analysis 5. RR 0.88, 95% CI 0.70 to 1.10 | Sensitivity analysis 5. MD -1.84, 95% CI -4.40 to 0.72 |
|  |  | Admission avoidance and early discharge | Sensitivity analysis 5. RR 1.24, 95% CI 0.50 to 3.05 | Sensitivity analysis 5. RR 1.32, 95% CI 0.90 to 1.95 | Sensitivity analysis 5. MD 3.10, 95% CI 1.81 to 4.39 |
|  | | | | | |
| **Grouping all interventions into the broader inpatient-level care at home (post hoc exploratory analyses)** | | | **Mortality** | **Hospital readmission** | **Length of stay in days** |
| RCT data | | | RR 0.91, 95% CI 0.76 to 1.09 | RR 0.92, 95% CI 0.77 to 1.11 | MD 1.55, 95% CI 0.25 to 2.84 |
| Non-randomised data | | | RR 0.51, 95% CI 0.29 to 0.91 | RR 0.72, 95% CI 0.55 to 0.95 | MD -1.74, 95% CI -2.52 to -0.95 |

Notes. * Results presented used RCT data unless otherwise where was specified in this table.

Sensitivity analysis 1 assumed no technology to be low technology.

Sensitivity analysis 2 used fixed effect model rather than random effects model.

Sensitivity analysis 3 excluded studies at overall high risk of bias.

Sensitivity analysis 4 merged general inpatient-level care and extended multidisciplinary inpatient care.

Sensitivity analysis 5 classified interventions into models of admission avoidance, early discharge or both.

**Table S2. Characteristics of the included studies**

| **Study identity** | **Publication type** | **Country** | **Study design** | **Allocation approaches** | **Health conditions** | **Sample size** | **No. of participants (Intervention)** | **No. of participants (Control)** | **Mean age (SD), years** | **Male (%)** | **Interventions** | **Control** | **Outcomes reported** | **Follow up** | **Methods are used to control for confounding in non-RCT studies** | **Overall risk of bias** |
| --- | --- | --- | --- | --- | --- | --- | --- | --- | --- | --- | --- | --- | --- | --- | --- | --- |
| ACTRN12621000692831 [30] | Registry record | Australia | RCT | Randomisation | Acute uncomplicated diverticulitis | 40 | NA | NA | NA | NA | rpavirtual (Virtual Healthcare) | inpatient care | NA | 1 month | NA | Not relevant |
| Caplan 1999 [31]; Caplan 2005 [32]; Board 2000 [33] | Journal paper | Australia | RCT | Randomisation | Acute medical conditions | 100 | 51 | 49 | 70 | 45 (45%) | Hospital in the home | Hospital care | Mortality, readmission, cost effectiveness, length of stay, adverse events | 1 month, 6 months | NA | Unclear |
| Corwin 2005 [34] | Journal paper | New Zealand | RCT | Randomisation | Cellulitis | 200 | 101 | 99 | 51.5 (20) | 131 (67.5%) | IV antibiotics at home | IV antibiotics in hospitals | Readmission, length of stay | 1 month | NA | Unclear |
| Kalra 2000 [35]; Patel 2003 [66] | Journal paper | UK | RCT | Randomisation | Acute stroke | 457 | 153 | 304 | NA | 234 (52.3%) | Home stroke care | Stroke unit and stroke team (hospital based) | Mortality, cost effectiveness | 3, 6, 12 months | NA | Low |
| Hendricks 2011 [36]; Talcott 2011 [37] | Journal paper | USA | RCT | Randomisation | Febrile neutropenia | 121 | 50 | 71 | Mean 47 (range 20 to 81) | 52 (46%) | Home care (early discharge for IV antibiotics) | Hospital care | Adverse events, cost effectiveness | NA | NA | Unclear |
| Adler 1978 [38] | Journal paper | UK | RCT | Randomisation | Inguinal hernia or varicose veins (surgeries for these conditions) | 224 | 117 | 107 | 18 to 64 | 124 (55.4%) | Short stay (early discharge) | Long stay (hospital care) | Adverse events, cost effectiveness | 7 days | NA | Unclear |
| Booth 2004 [39] | Journal paper | UK | RCT | Randomisation | Coronary artery bypass grafting | 97 | 65 | 32 | NA | NA | Early discharge | Hospital care | Readmission, length of stay, cost effectiveness | 3 months | NA | Unclear |
| Cotton 2000 [40] | Journal paper | UK | RCT | Randomisation | COPD exacerbations | 81 | 41 | 40 | 66.8 (1.8) | 35 (43.2%) | Early discharge next working day with home treatment supported by respiratory nurses | Hospital care | Mortality, readmission, length of stay | 2 months | NA | Unclear |
| Díaz Lobatoa 2005 [41] | Journal paper | Spain | RCT | Randomisation | COPD exacerbations | 40 | 20 | 20 | 66 (9) | 34 (85%) | Early discharge home hospitalization | Hospital care | Length of stay | 1 month | NA | High |
| Skwarska 2000 [42] | Journal paper | UK | RCT | Randomisation | COPD exacerbations | 184 | 122 | 62 | Median 69 (range 39 to 86) | 87 (47.3%) | Supported discharge | Hospital care | Mortality, readmission, cost effectiveness | 2 months | NR | Unclear |
| Gallier 2021 [43] | Preprint | UK | Retrospective cohort study using electronic health record dataset | Unspecified, participants were grouped into those admitting for longer than 24 hours, and those being discharged | COVID-19 | 571 | 325 | 246 | Median 51 (IQR 37 to 68) | 262 (45.9%) | Covid virtual ward (discharge within 24 hours of presentation) | Hospitalisation | Mortality, length of stay | 1 month | Univariate analysis, no adjustment | Critical risk of bias |
| NCT05920304 [44] | Registry record | Denmark | RCT | Randomisation | General unspecified acute medical conditions | NA | NA | NA | NA | NA | virtual Hospital at Home | Conventional hospitalisation | Mortality, readmission, length of stay, adverse events, cost effectiveness | 7 days, 1 month, 3 months | NA | Not relevant |
| NCT05256303 [45] | Registry record | USA | RCT | Randomisation | General acute medical conditions | NA | NA | NA | NA | NA | Home Hospital care | Traditional Hospital care | Mortality, readmission, length of stay, adverse events, cost effectiveness | 2 months | NA | Not relevant |
| NCT03490084 [46] | Registry record | France | Prospective cohort study | Unspecified | Elderly patients with multiple myeloma | NA | NA | NA | NA | NA | One day hospitalisation and 3 weekly chemotherapy at home | Day hospitalisation exclusively (4 weekly chemotherapy) | Mortality, cost effectiveness | 6 months, 12 months | NA | Not relevant |
| Aibar 2013 [47] | Conference abstract | unspecified | unspecified | NA | cancer | 366 | 247 | 119 | NA | NA | hospital at home | hospitalisation | Mortality, length of stay | NA | NA | No information |
| Aimonino 2002 [48] | Conference abstract | Italy | RCT | Randomisation | Advanced dementia | 82 | NA | NA | NA | NA | GHHS treatment | hospital | Mortality | NA | NA | High |
| Aimonino Ricauda 2008 [49,50] | Journal paper | Italy | RCT | Randomisation | COPD exacerbation | 104 | 52 | 52 | 79.6 (3.2) | 68 (65.4%) | geriatric home hospitalisation service | general ward | Mortality, hospital readmission, cost effectiveness, length of stay | 6 months | NA | Unclear |
| Ansari 2009 [51] | Journal paper | UK | prospective cohort | Forming groups according to where patients were cared | AECOPD (COPD exacerbations) | 90 | 60 | 30 | 74 (8.6) | 47 (52.2%) | Urgent Care Team (UCT) of nurse practitioners in communities | hospital care | Mortality | 2 to 3 months | Univariate analysis only | Serious |
| Federman 2018 [52], Augustine 2021 [53] | Journal paper | US | prospective cohort, and its secondary analysis | Forming groups according to the availability of intervention treatments and patients' willingness to use interventions | 19 acute medical conditions | 507 | 295 | 212 | 74.6 (15.7) | 159 (31.4%) | HaH | hospital | Mortality, hospital readmission, adverse events, length of stay | 1 month | Inverse probability of treatment weights used, considering the covariates of age, sex, race and ethnicity, education, self-reported health status, insurance type, admission diagnosis, caregiver status, and paid caregiver status. Regression analysis used | Moderate |
| Bagust 2002 [54], Sartain 2002 [55], ISRCTN11421664 [56] | Journal paper, registry | UK | RCT | Randomisation | hospitalised moderately ill children with: breathing difficulties, diarrhoea with or without vomiting, or a feverish illness. | 399 (effectiveness evaluation), 300 (economic evaluation) | 210 | 189 | 2.1 | 240 (60.2%) | Acute paediatric hospital at home scheme (a nursing scheme) | paediatric wards of a district general hospital | Hospital readmission, cost effectiveness, length of stay | 3 months | NA | Unclear |
| Cai 2017 [57] | Journal paper | US | Retrospective cohort study | Forming groups according to electronic dataset records of using interventions or not; propensity score matching used | general conditions | 421 | 99 | 322 | median 73.32 for hospital at home, 68.56 for the control | 405 (96.2%) | hospital at home | hospitalisation | Mortality, hospital readmission, length of stay | 6 months | Propensity score-matching used, considering the covariates of age, the number of medications | Serious |
| Cai 2018 [58] | Journal paper | US | Retrospective cohort study based on Medicare and VA data | Forming groups according to electronic dataset records of using interventions or not; propensity score matching used | any conditions | 829 | 137 | 692 | 67.2 | NA | Cincinnati HIH | traditional inpatient care | Mortality, hospital readmission, cost effectiveness | 1 month after discharge (mortality and readmission), 6 months (mortality) | Propensity score-matching used, considering the covariate of age | Serious |
| Cai 2021 [59] | Journal paper | US | Retrospective cohort study based on Medicare and VA data | Forming groups due to the geographical restriction (or availability of interventions in different geographical areas) that is suitable to overcome selection bias. Propensity score matching used | general conditions | 405 | 108 | 297 | 66.7 (0.83) | 399 (98.5%) | transfer component of a Veterans Affairs (VA) Hospital in Home program (T-HIH) | hospitalisation | Mortality, hospital readmission, cost effectiveness, length of stay | 3 months | NA | Serious |
| Campbell 2001 [60] | Journal paper | UK | Cohort study, with discrete event simulation used for cost minimisation analysis | Forming groups according to patients' consent to the intervention treatments | medical and orthopaedic surgical patients | 51 | 30 | 21 | 81.7 | 11 (21.6%) | HaH | hospital inpatient care | Mortality, hospital readmission, cost effectiveness, adverse events | < 3 months post-discharge | Discrete event simulation used for cost analysis | Critical |
| Cryer 2012 [61] | Journal paper | US | Retrospective cohort study based on Medicare and VA data | Forming groups according to the use of intervention treatments with matched controls identified from administrative database | exacerbations of congestive heart failure, chronic obstructive pulmonary disease, community- acquired pneumonia, cellulitis, deep venous thrombosis, pulmonary embolism, complicated urinary tract infection or urosepsis, nausea and vomiting, and dehydration | 2728 | 323 | 2405 | 79.1 (8.7) | 1197 (43.9%) | Presbyterian hospital at home | hospital care | Mortality, hospital readmission, cost effectiveness, length of stay | 1 month after discharge | NA | Critical |
| Davies 2000 [62,63] | Journal paper | UK | RCT | Randomisation | COPD exacerbation | 150 | 100 | 50 | 70 (8) | 75 (50%) | Home care | Hospital care | Mortality, hospital readmission | 3 months | NA | Unclear |
| Echevarria 2018 [64,65]; ISRCTN29082260 [67] | Journal paper; Registry record | UK | RCT | Randomisation | COPD exacerbation | 118 | 60 | 58 | 70 (9.6) | 56 (47.5%) | hospital at home (HAH) | Usual inpatient care | Mortality, hospital readmission, cost effectiveness, length of stay | 3 months | NA | Unclear |
| Escartin 2017 [68] | Journal paper | Spain | retrospective analysis of the data collected in a patient registry | Forming groups according to if clinicians referred intervention treatments to patients | mild or moderate acute calculous cholecystitis | 915 | 147 | 768 | 70 (16) | 577 (63.1%) | hospital at home | hospital care | Mortality, hospital readmission, adverse events, length of stay | 1 month for readmission | Covariates of age, ASA grade III-IV, severity of acute cholecystitis, types of treatments considered; one-way analysis of variance used only | Critical |
| Esmond 2006 [69] | Journal paper | UK | quasi-experimental study | Forming groups according to patient choices | acute respiratory exacerbation of cystic fibrosis | 30 | 15 | 15 | 24.5 (5.7) | 15 (50%) | i.v. antibiotics at home | i.v. antibiotics in hospital | NA | NA | Age considered | Critical |
| Gonzalez Barca 2006 [70] | Journal paper | Spain | unspecified, cohort of hospital at home patients was identified and then the control group using hospital based care was matched. | Forming groups according to patients' eligibility to intervention treatments | acute respiratory patients | 75 | 25 | 50 | 74.7 (9.7) | NA | hospital at home | hospital care | Mortality, hospital readmission, length of stay | 3 months | Sex matching; considering age, FEV1, FEV1/FVC, PaO2 and other measures that reflect severity of respiratory conditions, Charlson comorbidity; univariate analysis methods used only | Critical |
| Gruss 2013 [71] | Conference abstract | Uruguay | retrospective analysis | NA | Community-acquired pneumonia | 152 | 49 | 94 | 59.7 (19.3) | 59 (38.8%) | hospital at home | hospital care | Cost effectiveness, length of stay | NA | NA | No information |
| Harris 2005 [72,73] | Journal paper | New Zealand | RCT | Randomisation | acute medical conditions | 285 | 143 | 142 | 80 | Majority of women | hospital at home (HAH) | Hospital care | Mortality, hospital readmission, cost effectiveness, length of stay | 3 months | NA | High |
| Hatziagorou 2015 [74] | Conference abstract | unspecified | prospective clinical study | NA | cystic fibrosis | 35 | 20 | 15 | 12.6 (7) | 20 (57.1%) | home iv antibiotic | hospital iv antibiotic | Adverse events, cost effectiveness | NA | NA | No information |
| Hensher 1996 [75] | Journal paper | UK | cost minimization analysis | Forming groups according to catchment areas where patients lived | orthopaedic conditions | 851 HaH, unknown for the control | 851 | NA | NA | NA | HaH | Orthopaedic ward | Cost effectiveness, length of stay | NA | Univariate analysis only | Not relevant |
| Herranz 2022 [76] | Journal paper | spain | prospective cohort studies aiming for comparative effectiveness evaluations | NA, propensity score matching used | non-surgical patients | 274 | 137 | 137 | 72 (14) | 159 (58%) | hospital at home (HAH) | Usual Care (UC) | Mortality, hospital readmission, cost effectiveness, length of stay | 1 month | Propensity score matching, considering age; gender; number of admissions during the previous year; patients healthcare costs across the health system in the previous year; patients population-based risk (Adjusted Morbidity Groups GMA) scoring; weighted multiple regression models used | Moderate |
| Ince 2014 [77,78] | Journal paper | Turkey | RCT | Randomisation | mild non-alcoholic acute pancreatitis | 84 | 42 | 42 | 54 (18) | 29 (34.5%) | Home group | Hospital group | Hospital readmission, cost effectiveness | 1 month | NA | Unclear |
| ISRCTN36101176 [79] | Registry record | Norway | RCT | Randomisation | COPD exacerbations | 180 planned | NA | NA | NA | NA | Hospital-at-home | Hospital | NA | 12 months | NA | Not relevant |
| ISRCTN36662318 [80] | Registry record | Chile | RCT | Randomisation | Adult inpatients with moderate risk diseases | 102 planned | NA | NA | NA | NA | Hospital at home | standard care in the hospital | NA | NA | NA | Not relevant |
| Jakobsen 2013, 2015 [81-83] | Journal paper | Denmark | RCT | Randomisation | COPD acute exacerbations | 57 | 29 | 28 | 70.5 (10.9) | 22 (38.6%) | Virtual Hospital | hospital care | Mortality, hospital readmission, adverse events | 1, 3 and 6 months after discharge | NA | Unclear |
| Jester 2003 [84-86] | Journal paper | UK | quasi-experimental study | Forming groups according to patients' preference | hip and knee replacement | 109 | 64 | 45 | 74 (range 64 to 86) | NA | early discharge to HaH on the fourth postoperative day | traditional in-patient rehabilitation within an orthopaedic unit | Adverse events, cost effectiveness | 6 months | Univariate analysis only | Critical |
| Leff 2005 [87-91] | Journal paper | US | Prospective quasi-experiment | Forming groups according to separate time periods when patients were admitted | Acutely Ill Older Patients | 455 | 169 | 286 | 77.3 (6.7) | 276 (60.7%) | hospital at home | hospital care | Mortality, hospital readmission, adverse events, length of stay | 0.5 and 2 months | Logistic regression used, adjusted for age, gender, APACHE II score, and study site | Critical |
| Levine 2018 [92] | Journal paper | US | RCT | Randomisation | any infection, heart failure exacerbation, COPD exacerbation, or asthma exacerbation | 21 | 9 | 11 | median 65 (IQR 28) for hospital at home, 60 (29) for the control | 10 (47.6%) | Home Hospital | usual care | Hospital readmission, length of stay | 1 month after discharge | NA | Unclear |
| Levine 2019 [93] | Journal paper | United States | RCT | Randomisation | Acute Conditions (Primary or possible diagnosis of any infection, heart failure exacerbation, COPD exacerbation, asthma exacerbation, chronic kidney disease requiring diuresis, diabetes and its complications, gout exacerbation, hypertensive urgency, previously diagnosed atrial fibrillation with rapid ventricular response, anticoagulation needs (e.g., venous thromboembolism)) | 91 | 43 | 48 | Average 80 (IQR 19) for hospital at home, 72 (23) for the control | 58 (63.7%) | Home Hospital | Usual care | Hospital readmission, cost effectiveness, length of stay | 1 month | NA | Unclear |
| Mendoza 2009 [94] | Journal paper | Spain | RCT | Randomisation | Decompensated heart failure | 71 | 37 | 34 | 79 (6.3) | 42 (59.2%) | hospital at home | cardiology ward | Mortality, hospital readmission, cost effectiveness, length of stay | 1, 3, 6, 12 months | NA | Unclear |
| Morgan 2019 [95] | Conference abstract (published in a journal) | United Kingdom | Non-RCT | NA | COPD exacerbation | 132 | 24 | 108 | median 77 | NA | hospital at home | admitted to hospital | Mortality, hospital readmission | 12 months | NA | Critical |
| NCT04330378 [96] | Registry record | Singapore | Cohort studies aiming for comparative effectiveness evaluations | Forming groups according to the capacity of enrolling patients into the intervention treatments | acute conditions | 441 planned | NA | NA | NA | NA | hospital at home | Usual in-hospital care | NA | NA | NA | Not relevant |
| NCT05360914 [97] | Registry record | Denmark | RCT | Randomisation | Acute ill patients | 849 | NA | NA | NA | NA | hospital at home | standard hospital admission | NA | NA | NA | Not relevant |
| O'Cathain 1994 [98] | Journal paper | United Kingdom | Non-RCT | Forming groups according to the use of intervention treatments | fractured neck of femur | 110 | 76 | 34 | 76.8 (9.9) | 15 (13.6%) | hospital at home | Hospital | Mortality, hospital readmission, cost effectiveness, length of stay | NA | Univariate analysis used only, after controlling for confounders such as gender, age, mental functioning and pre-injury mobility | Critical |
| Ojoo 2002 [99] | Journal paper | UK | RCT | Randomisation | COPD acute exacerbations | 60 | 30 | 30 | 69.9 | 31 (51.7%) | hospital at home | inpatient care | Mortality, hospital readmission | 3 months | NA | High |
| Oterino-de-la-Fuente 1998 [100,101] | Journal paper | spain | Cohort studies aiming for comparative effectiveness evaluations | Forming groups by matching controls | General acute and non-acute conditions | 296 | 148 | 148 | 15-44 years: 9.5%; 45-64 years: 25.7%; 65-79 years: 44.6%; >=80 years: 20.3% in hospital at home, 15-44 years: 9.5%; 45-64 years: 25.7%; 65-79 years: 44.6%; >=80 years: 20.3% in the control | 158 (53.3%) | hospital at home | Hospital | Mortality, hospital readmission, adverse events, cost effectiveness, length of stay | NA | Univariate analysis used only, matching controls according to the disease group, age and sex | Critical |
| Pouw 2018 [102], NTR6581 [103] | Published protocol and registry record | Netherlands | RCT | Randomisation | cognitively impaired older patients who are in need of acute hospital care. | 143 planned | NA | NA | NA | NA | hospital at home | usual hospital care | NA | 3 and 6 months | NA | Not relevant |
| Ricauda et al 2004 [104], Ahrens 2004 [105], Ricauda 2005 [106] | Journal paper | Italy | RCT | Randomisation | ischemic stroke | 120 | 60 | 60 | 82 | 54 (45%) | home treatment from a geriatric home hospitalization service (GHHS) | general medical ward | Mortality, cost effectiveness, length of stay | 6 months | NA | Unclear |
| Richards 1998 [107], Coast 1998 [108], Gunnell 2000 [109,110] | Journal paper,Registry record | UK | RCT | Randomisation | hospitalised but medically stable elderly patients | 241 | 160 | 81 | median 79 (IQR 72-84) in hospital at home, 79 (74-84) in the control | 74 (30.7%) | hospital at home | hospital care | Mortality, cost effectiveness, length of stay | 3 months | NA | Unclear |
| Rodriguez-Cerrillo 2013 [111] | Journal paper | Spain | Cohort studies aiming for comparative effectiveness evaluations | Forming groups according to atients' preference to use of intervention treatments | uncomplicated diverticulitis | 52 | 34 | 19 | 77.7 | 9 (17.3%) | hospital at home | hospital care | Cost effectiveness, length of stay | NA | NA | Critical |
| Rousseau 2019 [112] | Conference abstract | US | Cohort studies aiming for comparative effectiveness evaluations | NA | pneumonia | 99 | 65 | 34 | NA | NA | hospital at home | hospitalisation | Mortality, hospital readmission | NA | NA | Serious |
| Saenger 2022 [113] | Journal paper, cost analysis using part of Federman 2018 data | US | retrospective observational cohort | Forming groups according to the availability of intervention treatments and patients' willingness to use interventions | conditions requiring inpatient admission | 302 | 201 | 101 | 79.3 (13.3) | 91 (30.1%) | Hospital at home | inpatient care | Cost effectiveness | 1 month | NA | Not relevant |
| Sequerios 2013 [114] | Conference abstract | Unspecified | Non-RCT | NA | cystic fibrosis (CF) pulmonary exacerbations | 58 | NA | NA | NA | NA | hospital at home | hospital care | NA | NA | NA | No information |
| Shepperd 1998 [115-118] | Journal paper,Registry record | UK | RCT | Randomisation | hip replacement, knee replacement, hysterectomy, COPD, elderly patients with a mix of medical con­ditions | 538 | 163 | 275 | 60 | 119 (22.1%) | hospital at home | hospital care | Mortality, hospital readmission, cost effectiveness | 3 months | NA | Unclear |
| Shepperd 2017 [119]; Shepperd 2021 [120]; Singh 2022 [121], ISRCTN60477865 [122] | Journal paper,Registry record | UK | RCT | Randomisation | older people with markers of frailty or prior dependence | 1055 | 687 | 345 | 83.3 (7) | 407 (38.6%) | geriatrician-led admission avoidance hospital at home | hospital care | Mortality, hospital readmission, cost effectiveness, length of stay | 12 months | NA | Unclear |
| Soones 2016 [123] | Conference abstract | US | Non-randomized, observational study comparing MACT participants with concurrent controls | Forming groups according to the availability of intervention treatments and patients' willingness to use interventions | general acute conditions | 90 | 34 | 56 | 71 | NA | Mobile Acute Care Team | hospital care | Hospital readmission, length of stay | NA | NA | No information |
| Summerfelt 2015 [124] | Journal paper | US | Prospective, nonrandomized, quasi-experiment | Forming groups according to specific time periods when the intervention treatments were accessible (i.e., within or outside of service hours) | exacerbation of chronic obstructive pulmonary disease (COPD) or congestive heart failure (CHF), deep vein thrombosis (DVT), asthma, or community- acquired pneumonia | 102 | 50 | 52 | 63.5 (14.5) | 44 (43.1%) | hospital at home | hospital care | Mortality, hospital readmission, length of stay | 3 months | Adjusted logistic regression, controlling for the covariates of age, acuity levels, sociodemographic items, comorbidity index | Moderate |
| Tibaldi 2009 [125, 126] | Journal paper,Registry record (NCT00623571) | Italy | RCT | Randomisation | acute decompensation of chronic heart failure | 101 | 48 | 53 | 81.1 (5.1) | 52 (51.5%) | Geriatric Home Hospitalization Services (GHHS) | general medical ward (GMW) | Mortality, hospital readmission, cost effectiveness, length of stay | 6 months | NA | Unclear |
| Tibaldi 2013 [127] | Journal paper | Italy | RCT | Randomisation | Elderly patients with acute decompensation of severe chronic heart failure | 52 | 26 | 26 | 81 | 26 (50%) | Geriatric Home Hospitalization Services (GHHS) | general medical ward (GMW) | Mortality, hospital readmission, length of stay | 1 month | NA | Unclear |
| Toral-Lopez 2017 [128] | Journal paper | Spain | Non-RCT | Forming groups according to the distance to hospitals and consent | preterm infants | 86 | 46 | 40 | Infants | NA | early discharge intervention | hospitalisation | Length of stay | NA | NA | Critical |
| Tsiachristas 2019 [129] | Journal paper | UK | retrospective propensity score-matched analysis using administrative data | NA | unspecified | 22610 | 3633 | 18977 | 78.5 | 9732 (43%) | geriatrician-led admission avoidance hospital-at-home services | hospital care | Mortality, cost effectiveness | 6 months after discharge | Propensity score matching used, considering the covariates of age, Scottish Index of Multiple Deprivation, Long-term conditions; regression used | Critical |
| Utens 2010, 2013, 2014 [130-132] | Journal paper | Netherlands | RCT | Randomisation | COPD exacerbations | 139 | 70 | 69 | 68.1 (10.8) | 53 (38.1%) | hospital at home | hospital care | Mortality, hospital readmission | 3 months | NA | Unclear |
| Vianello 2013 [133] | Journal paper | Italy | RCT | Randomisation | Neuromuscular Disease Patients with severe respiratory tract infection | 53 | 26 | 27 | 45.7 (20.1) | 41 (77.4%) | HaH | hospital | Mortality, cost effectiveness | 3 months | NA | Unclear |
| Wilson 1998 [134], Wilson 1999 [135], Wilson 2002 [136,137], Jones 1999 [138] | Journal paper,Grant report | UK | RCT | Randomisation | acute conditions | 199 | 102 | 97 | median 84 (range 77-­89) in hospital at home, 84 (77-89) in the control | 57 (28.6%) | Leicester hospital at home scheme | hospital care | Mortality, hospital readmission, cost effectiveness, length of stay | 3 months | NA | Unclear |
| Yao 2022 [139] | Journal paper of a protocol | US | RCT | Randomisation | acutely ill adult patients | 360 planned | NA | NA | NA | NA | Advanced Care at Home | hospital care | NA | 1 month | NA | Not relevant |

**Table S3. Risk of bias assessment results for RCTs**

| Studisses | Supporting statement - Sequence generation | Supporting statement - Allocation concealment | Supporting statement - Blinding of participants and personnel | Supporting statement - Blinding of outcome assessment | Supporting statement - Incomplete data | Supporting statement - Selective outcome reporting | Other issues | Overall RoB | Comments |
| --- | --- | --- | --- | --- | --- | --- | --- | --- | --- |
| ACTRN12621000692831 | NI | NI | NI | NI | NI | NI | NI | NI | Registry record |
| Adler 1978 | Unclear (no information) | Unclear (no information) | High (unlikely to blind) | Unclear (no information) | Low (no missing data) | Low (no concern) | Low (no concern) | Unclear |  |
| Aimonino 2002 | Unclear | Unclear | Unclear | Unclear | Unclear | Unclear | Unclear | High | Conference abstract |
| Aimonino Ricauda 2008 | Low (computer-generated random numbers) | Unclear (sealed, numbered envelope) | High (unlikely to blind) | Low (blinded outcome assessment) | Low (low missing rate) | Low (no concern) | Low (no concern) | Unclear |  |
| Bagust 2002, Sartain 2002, ISRCTN11421664 | Unclear (no details) | Low (sealed numbered and opaque envelope) | High (unlikely to blind) | Unclear (no information) | Low (low missing rate, 10/399) | Low (no concern) | Low (no concern) | Unclear |  |
| Booth 2004 | Unclear (no information) | Unclear (no information) | High (unlikely to blind) | Unclear (no information) | Low (no missing data) | Low (no concern) | Low (no concern) | Unclear |  |
| Caplan 2000 | Low (computer-generated random numbers coded into sealed envelopes) | Unclear (sealed envelopes) | High (unlikely to blind) | Unclear (no information) | Low (no missing data) | Low (no concern) | Low (no concern) | Unclear |  |
| Corwin 2005 | Low (randomisation list produced by SAS code… using randomly allocated block sizes with a maximum of 20) | Low (allocated by phoning an off-site coordinator who kept the randomisation list) | High (unlikely to blind) | Unclear (no information) | Low (low missing rate, 3%) | Low (no concern) | Low (no concern) | Unclear |  |
| Cotton 2000 | Unclear (random numbers) | Low (telephoning an independent staff) | High (unlikely to blind) | Unclear (no information) | Low (ITT analysis) | Low (no concern) | Low (no concern) | Unclear |  |
| Davies 2000 | Unclear (no details) | Unclear (blinded sealed envelopes) | High (unlikely to blind) | Unclear (no information) | Low (ITT analysis used) | Low (no concern) | Low (no concern) | Unclear |  |
| Díaz Lobatoa 2005 | Unclear (no information) | Unclear (no information) | High (unlikely to blind) | High (open trial) | Low (no missing data) | Unclear (length of stay outcome reported in results but not in methods) | Low (no concern) | High |  |
| Echevarria 2018 (ISRCTN29082260) | Low (1:1 randomisation via minimisation undertaken by an external, independent agency, sealedenvelope.com) | Low (1:1 randomisation via minimisation undertaken by an external, independent agency, sealedenvelope.com) | High (unlikely to blind) | Unclear (no information) | Low (2/120, low missing rate) | Low (no concern) | Low (no concern) | Unclear |  |
| Harris 2005 | Low (computer-generated randomisation) | Low (randomisation, independent of all parties and accessible by telephone 24 hours per day) | High (unlikely to blind) | High (no blinding) | Low (no concern) | Low (no concern) | Low (no concern) | High |  |
| Hendricks 2011; Talcott 2011 | Low (computer-generated random assignments using blocks) | Unclear (no information) | High (unlikely to blind) | Low (blinded assessment) | Low (low missing rate, 8/121) | Low (no concern) | Low (no concern) | Unclear |  |
| Ince 2014 | Low (computer randomisation) | Unclear (not reported) | High (unlikely to blind) | Unclear (no information) | Low (no concern) | Low (no concern) | Low (no concern) | Unclear |  |
| ISRCTN36101176 | NI | NI | NI | NI | NI | NI | NI | NI | Trial registry records only |
| ISRCTN36662318 | NI | NI | NI | NI | NI | NI | NI | NI | Trial registry records only |
| Jakobsen 2013, 2014, 2015 | Low (computer-generated tables) | Low (sequentially numbered, sealed and opaque envelopes) | High (unlikely to blind) | Low (blinded outcome assessment) | Low (ITT analysis used) | Unclear (unclear reporting of GP visits outcome) | Low (no concern) | Unclear |  |
| Kalra 2000 | Low (computer generated with random numbers) | Low (allocation codes held in a central office and contacting via telephone for allocations) | High (unlikely to blind) | Low (assessors independent of the health care provided and unaware of treatment allocation) | Low (low missing rate, 4%, ITT analysis) | Low (no concern) | Low (no concern) | Low |  |
| Levine 2018 | Unclear (block randomisation) | Unclear (sealed opaque envelopes) | High (unlikely to blind) | Unclear (no information) | Low (1/21 missing rate) | Low (no concern) | Low (no concern) | Unclear |  |
| Levine 2019 | Low (block randomisation, SAS generated sequence) | Unclear (sealed opaque envelopes) | High (unlikely to blind) | Unclear (no information) | Low (no missing) | Low (no concern) | Unclear (an early stopped trial) | Unclear |  |
| Mendoza 2009 | Unclear (no details) | Low (externally generated sequence hidden until patient consented) | High (unlikely to blind) | Unclear (no information) | Unclear (9/80 missing rate) | Low (no concern) | Unclear (an early stopped trial) | Unclear |  |
| NCT05360914 | NI | NI | NI | NI | NI | NI | NI | NI | Registry record |
| NCT05920304 | NI | NI | NI | NI | NI | NI | NI | NI | Registry record |
| NCT05256303 | NI | NI | NI | NI | NI | NI | NI | NI | Registry record |
| Ojoo 2002 | Unclear (no details) | Unclear (sealed envelopes) | High (unlikely to blind) | Unclear (no information) | High (6/60 missing rate, but 2 exclusions due to readmisions that are an outcome) | Low (no concern) | Low (no concern) | High |  |
| Pouw 2018, NTR6581 | NI | NI | NI | NI | NI | NI | NI | NI | Published protocol and registry record |
| Ricauda et al 2004, Ahrens 2004, Ricauda 2005 | Low (block randomisation; a block size of 2 patients) | Unclear (no details) | High (single blinding that was for outcome assessments) | Low (blinded outcome assessment) | Low (ITT analysis used) | Low (no concern) | Low (no concern) | Unclear |  |
| Richards 1998, Coast 1998, Gunnell 2000 | Unclear (block randomisation) | Unclear (sealed envelopes) | High (unlikely to blind) | Unclear (no details) | Low (ITT analysis used) | Low (no concern) | Low (no concern) | Unclear |  |
| Shepperd 1998 | Low (computer-generated randomisation) | Unclear (sealed opaque envelopes) | High (unlikely to blind) | Unclear (no details) | Low (no missing) | Low (no concern) | Low (no concern) | Unclear |  |
| Shepperd 2017; Shepperd 2021; Singh 2022, ISRCTN60477865 | Low (computer-generated randomisation) | Unclear (no information) | High (unlikely to blind) | Unclear (no details) | Low (ITT analysis used) | Low (no concern) | Low (no concern) | Unclear |  |
| Skwarska 2000 | Low (computer generated random numbers) | Unclear (no information) | High (unlikely to blind) | Unclear (no information) | Low (no missing data) | Unclear (outcomes unspecified) | Low (no concern) | Unclear |  |
| Tibaldi 2009 | Low (computer-generated random numbers) | Unclear (numbered sealed envelopes) | High (unlikely to blind) | Low (blinded outcome assessment) | Low (low missing rate) | Low (no concern) | Low (no concern) | Unclear |  |
| Tibaldi 2013 | Low (computer-generated random numbers) | Unclear (numbered sealed envelopes) | High (unlikely to blind) | Unclear (no details) | Low (no missing) | Low (no concern) | Low (no concern) | Unclear | To check with Jo if this could be included |
| Utens 2010, 2013, 2014 | Low (computer-generated randomisation) | Unclear (no information) | High (no blinding) | Unclear (no information) | Low (ITT analysis used) | Low (no concern) | Low (no concern) | Unclear |  |
| Vianello 2013 | Unclear (no details) | Unclear (blinded sealed envelopes) | High (unlikely to blind) | Unclear (no information) | Low (no concern) | Low (no concern) | Low (no concern) | Unclear |  |
| Wilson 1998, Wilson 1999, Wilson 2002, Jones 1999 | Low (a block randomisation with block size 10) | Low (consecutively numbered sealed opaque envelopes) | High (unlikely to blind) | Unclear (no information) | Low (ITT analysis used) | Low (no concern) | Low (no concern) | Unclear |  |
| Yao 2022 | NI | NI | NI | NI | NI | NI | NI | NI | Published protocol |

**Table S4. Risk of bias assessment results for non-randomised studies**

| Studies | Bias due to confounding | Bias in selection of participants into the study | Bias in classification of interventions | Bias due to deviations from intended interventions | Bias due to missing data | Bias in measurement of outcomes | Bias in selection of the reported result | Overall risk of bias |
| --- | --- | --- | --- | --- | --- | --- | --- | --- |
| Aibar 2013 | NI | NI | NI | NI | NI | NI | NI | NI |
| Ansari 2009 | Serious (appropriate analysis methods not used for controlling for the important confounders) | Low (no concern) | Low (no concern) | No information | Low (no concern) | Low (no concern) | Low (no concern) | Serious |
| Cai 2017 | Moderate (confounding expected but confounders were measured well and controlled for) | Low (no concern) | Low (no concern) | Low (no concern) | Low (no concern) | Low (no concern) | Low (no concern) | Moderate |
| Cai 2018 | Moderate (confounding expected but confounders were measured well and controlled for) | Serious (participants inappropriately excluded due to their high care costs) | Low (no concern) | Low (no concern) | Low (no concern) | Low (no concern) | Low (no concern) | Serious |
| Cai 2021 | Serious (post-intervention variable was controlled for, which is inappropriate) | Low (no concern) | Low (no concern) | Low (no concern) | Low (no concern) | Low (no concern) | Low (no concern) | Serious |
| Campbell 2001 | Serious (confounding not well addressed) | Critical (follow up and start of intervention not at the same time) | Low (no concern) | Low (no concern) | Low (no concern) | Moderate (unblinding) | Low (no concern) | Critical |
| Cryer 2012 | Critical (appropriate analysis methods not used) | Serious (follow up following discharge rather than the start of hospital at home) | Low (no concern) | Low (no concern) | Low (no concern) | Moderate (unblinding) | Low (no concern) | Critical |
| Escartin 2017 | Critical (appropriate analysis methods not used) | Moderate (selecting only mild or moderate cases into analysis whilst severe cases might be related to outcomes such as death) | Low (no concern) | Low (no concern) | Low (no concern) | Moderate (unblinding) | Low (no concern) | Critical |
| Esmond 2006 | Critical (appropriate analysis methods not used) | Low (no concern) | Low (no concern) | Moderate (2/30 participants used both intervention and control) | Low (no concern) | Moderate (unblinding) | Low (no concern) | Critical |
| Federman 2018, Augustine 2021 | Moderate (inverse probability weighting used for limiting bias from nonrandom assignment) | Moderate ( people who present out of hours differ from those presenting in office hours) | NI | Low (no concern) | Low (no concern) | Moderate (unblinding) | Low (no concern) | Moderate |
| Gallier 2021 | Critical (appropriate analysis methods not used) | Low (no concern) | Low (no concern) | Low (no concern) | Low (no concern) | Moderate (unblinding) | Low (no concern) | Critical |
| Gonzalez Barca 2006 | Critical (appropriate analysis methods not used) | Low (no concern) | Low (no concern) | Low (no concern) | Low (no concern) | Moderate (unblinding) | Low (no concern) | Critical |
| Gruss 2013 | NI | NI | NI | NI | NI | NI | NI | NI |
| Hatziagorou 2015 | NI | NI | NI | NI | NI | NI | NI | NI |
| Hensher 1996 | NI | NI | NI | NI | NI | NI | NI | NI |
| Herranz 2022 | Moderate (matching and appropriate analysis used) | Moderate | Low (no concern) | Low (no concern) | Low (no concern) | NI | Low (no concern) | Moderate |
| Jester 2003 | Critical (appropriate analysis methods not used for controlling for the important confounders) | No information | Low (no concern) | Low (no concern) | Low (no concern) | Moderate (unblinding) | Low (no concern) | Critical |
| Leff 2005 | Moderate (adjusted analysis used) | Low (no concern) | Low (no concern) | Critical (a large proportion of participants in HaH used acute hospital care) | Low (no concern) | Moderate (unblinding) | Low (no concern) | Critical |
| Morgan 2019 | NI | NI | NI | NI | NI | NI | NI | NI |
| NCT04330378 | NI | NI | NI | NI | NI | NI | NI | NI |
| O'Cathain 1994 | Critical (inappropriate analysis of confounding) | Critical (inappropriate exclusion of participants) | Low (no concern) | Low (no concern) | Low (no concern) | Moderate (unblinding) | Low (no concern) | Critical |
| Oterino-de-la-Fuente 1998 | Critical (inappropriate analysis of confounding) | Low (no concern) | Low (no concern) | Low (no concern) | Low (no concern) | Moderate (unblinding) | Low (no concern) | Critical |
| Rodriguez-Cerrillo 2013 | Critical (appropriate analysis methods not used) | No information | Low (no concern) | Low (no concern) | Low (no concern) | Moderate (unblinding) | Low (no concern) | Critical |
| Rousseau 2019 | Serious (appropriate analysis methods not used for controlling for the important confounders) | No information | Low (no concern) | NI | NI | NI | NI | Serious |
| Saenger 2022 | NI | NI | NI | NI | NI | NI | NI | NI (cost analysis) |
| Sequerios 2013 | NI | NI | NI | NI | NI | NI | NI | NI |
| Soones 2016 | NI | NI | NI | NI | NI | NI | NI | NI |
| Summerfelt 2015 | Moderate (adjusted analysis used) | Moderate (participants were recruited in slightly different periods) | Low (no concern) | Low (no concern) | Low (no concern) | Moderate (unblinding) | Low (no concern) | Moderate |
| Toral-Lopez 2017 | Critical (appropriate analysis methods not used) | Low (no concern) | Low (no concern) | Low (no concern) | Low (no concern) | Moderate (unblinding) | Low (no concern) | Critical |
| Tsiachristas 2019 | Moderate (confounding expected) | Critical (patient selection depending on outcome characteristics) | Low (no concern) | Low (no concern) | Low (no concern) | Moderate (unblinding) | Low (no concern) | Critical |

**Table S5. Summarises of intervention components**

| **Intervention groups** | **Studies** | **Care models** | **Health conditions** | **Interventions** | **Digital technology** | **Workforce** | **Clinical care pathway** | **Information and support provision** | **Clinical management system** |
| --- | --- | --- | --- | --- | --- | --- | --- | --- | --- |
| ***General inpatient-level care, delivered by hospital- or community-based health professionals, with high intensity technology involvement (7 specific interventions)*** |  |  |  |  |  |  |  |  |  |
| General inpatient-level care, delivered by hospital-based health professionals, with high intensity technology involvement | Herranz 2022 | Avoidance of hospital admission | Non-surgical patients | Hospital at home | High intensity - fully technology enabled | Hospital-based health professionals | Acute care (nurse home visiting for face to face activities) | NA | Electronic record system |
| General inpatient-level care, delivered by hospital-based health professionals, with high intensity technology involvement | Cryer 2012 | Avoidance of hospital admission, Early discharge | Acute medical conditions | Presbyterian hospital at home | Intermediate intensity - video conferencing, remote monitoring | Hospital-based health professionals (including telehealth nurse) | Acute care (nurse and physician home visiting for face to face activities, remote monitoring) | Patient empowerment | Electronic record system |
| General inpatient-level care, delivered by hospital-based health professionals, with high intensity technology involvement | NCT04330378 | Unspecified | Acute medical conditions | Hospital at home | Intermediate intensity - communication, remote monitoring | Hospital-based health professionals | Acute care (nurse and physician home visiting) | NA | NA |
| General inpatient-level care (virtual), delivered by hospital-based health professionals, with high intensity technology involvement | ACTRN 12621000692831 | Unspecified | Acute uncomplicated diverticulitis | rpavirtual (Virtual Healthcare) | Intermediate intensity - video conferencing, remote monitoring | Hospital-based health professionals (virtual ward doctors and nurses) | Acute care (virtual care, remote monitoring) | NA | NA |
| General inpatient-level care (virtual), delivered by hospital-based health professionals, with high intensity technology involvement | Jakobsen 2013, 2014, 2015 | Early discharge | COPD acute exacerbations | Virtual Hospital | Intermediate intensity - video conferencing, remote monitoring | Hospital-based health professionals (virtual ward doctor and nurse) | Speciality treatment (COPD treatment, virtual round/ remote monitoring) | Patient empowerment | NA |
| General inpatient-level care (virtual), delivered by hospital-based health professionals, with high intensity technology involvement | Gallier 2021 | Early discharge | COVID-19 | Covid virtual ward (discharge within 24 hours of presentation) | Intermediate intensity | Hospital based health professionals (remote virtual ward) | General care services | NA | Electronic record system |
| General inpatient-level care (virtual), delivered by hospital-based health professionals, with high intensity technology involvement | NCT05920304 | Early discharge | General unspecified acute medical conditions | virtual Hospital at Home | Intermediate intensity (communication and monitoring) | Hospital based health professionals (remote virtual ward, face to face activities) | General care services | NA | NA |
| ***General inpatient-level care, delivered by hospital- or community-based health professionals, with low intensity technology involvement (16 specific interventions)*** |  |  |  |  |  |  |  |  |  |
| General inpatient-level care, delivered by hospital based health professionals, with low intensity technology involvement | Ansari 2009 | Avoidance of hospital admission | COPD acute exacerbations | Urgent Care Team of nurse practitioners in communities | Low intensity - telephone | Hospital outreach nurse dominated (hospital visiting nurses) | Speciality treatment (COPD treatment, nurse home visiting) | NA | NA |
| General inpatient-level care, delivered by hospital based health professionals, with low intensity technology involvement | Bagust 2002, Sartain 2002, ISRCTN11421664 | Early discharge | Hospitalised ill children | Acute paediatric hospital at home scheme (a nursing scheme) | Low intensity - telephone | Hospital based health professionals (nurse dominated, hospital consultants supervised) | Nursing care (nurse home visiting) | Patient empowerment | NA |
| General inpatient-level care, delivered by hospital-based health professionals, with low intensity technology involvement | Hendricks 2011; Talcott 2011 | Early discharge | Febrile neutropenia | Home care (early discharge for IV antibiotics) | Low intensity | Hospital based health professionals | IV antibiotics at home | Non-patient empowerment | NA |
| General inpatient-level care, delivered by hospital based health professionals, with low intensity technology involvement | Toral-Lopez 2017 | Early discharge | Hospitalised ill children | Early discharge intervention | Low intensity – telephone | Hospital outreach nurse dominated | Nursing care (nurse home visiting) | NA | NA |
| General inpatient-level care, delivered by hospital-based health professionals, with low intensity technology involvement | Ince 2014 | Early discharge | Acute pancreatitis | Home group | Low intensity – telephone | Hospital-based health professionals | Nursing care (IV infusion, nurse home visiting for face to face activities) | Patient empowerment | NA |
| General inpatient-level care, delivered by hospital based health professionals, with low intensity technology involvement | Mendoza 2009 | Avoidance of hospital admission | Acute decompensation of chronic heart failure | Hospital at home | Low intensity – telephone | Hospital based health professionals | Acute care (nurse home visiting) | NA | NA |
| General inpatient-level care, delivered by hospital based health professionals, with low intensity technology involvement | Ojoo 2002 | Early discharge | COPD acute exacerbations | Hospital at home | Low intensity – telephone | Hospital outreach nurse dominated | Speciality treatment (respiratory treatment, nurse home visiting) | Patient empowerment | NA |
| General inpatient-level care, delivered by hospital-based health professionals, with low intensity technology involvement | Rodriguez-Cerrillo 2013 | Avoidance of hospital admission | Acute uncomplicated diverticulitis | Hospital at home | Low intensity - telephone | Hospital-based health professionals | IV antibiotics (nurse and physician home visiting for face to face activities) | NA | NA |
| General inpatient-level care, delivered by community based health professionals, with low intensity technology involvement | Corwin 2005 | Avoidance of hospital admission | Cellulitis | IV antibiotics at home | Low intensity - telephone | Community based health professionals (GP, community nurses, face to face activities) | IV antibiotics at home | NA | NA |
| General inpatient-level care, delivered by hospital based health professionals, with low intensity technology involvement | Utens 2010, 2013, 2014 | Early discharge | COPD acute exacerbations | Hospital at home | Low intensity - telephone | Hospital outreach nurse dominated | Speciality treatment (COPD treatment, nurse home visiting) | Patient empowerment | NA |
| General inpatient-level care, delivered by hospital-based health professionals, with low intensity technology involvement | Gonzalez Barca 2006 | Avoidance of hospital admission | Acute respiratory conditions | Hospital at home | Low intensity – telephone (remote instruction) | Hospital-based health professionals | Acute care (nurse and physician home visiting for face to face activities) | NA | NA |
| General inpatient-level care, delivered by hospital-based health professionals, with low intensity technology involvement | NCT05360914 | Avoidance of hospital admission | Acute medical conditions | Hospital at home | Low intensity - telephone/ video conferencing | Hospital-based health professionals | Acute care | Non-patient empowerment | NA |
| General inpatient-level care, delivered by hospital based health professionals, with low intensity technology involvement | Campbell 2001 | Avoidance of hospital admission, Early discharge | Medical and surgical patients | Hospital at home | Low intensity - telephone | Hospital based health professionals | Acute care (face to face activities) | NA | NA |
| General inpatient-level care, delivered by hospital-based health professionals, with low intensity technology involvement | Cotton 2000 | Early discharge | COPD exacerbations | Early discharge next working day with home treatment supported by respiratory nurses | Low intensity | Hospital based health professionals (face to face, nurse dominated) | General care services | NA | NA |
| General inpatient-level care, delivered by hospital-based health professionals, with low intensity technology involvement | Díaz Lobatoa 2005 | Early discharge | COPD exacerbations | Early discharge home hospitalization | Low intensity | Hospital based health professionals (face to face) | General care services | Patient empowerment | Paper based records |
| General inpatient-level care, delivered by hospital-based health professionals, with low intensity technology involvement | NCT05256303 | Unspecified | General acute medical conditions | Home hospital care | Low intensity - telephone | Hospital based health professionals (face to face activities) | General care services | NA | NA |
| ***General inpatient-level care, delivered by hospital- and community-based professionals, with high intensity technology involvement (2 interventions)*** |  |  |  |  |  |  |  |  |  |
| General inpatient-level care, delivered by hospital- and community-based professionals, with high intensity technology involvement | Summerfelt 2015 | Avoidance of hospital admission | Acute medical conditions | Hospital at home | High intensity - fully technology enabled | Hybrid team of hospital-based health professionals and GP | Acute and post acute care (nurse and physician home visiting for face to face activities, virtual ward/ remote monitoring, post acute care with GP) | Patient empowerment | NA |
| General inpatient-level care, delivered by hospital- and community-based professionals, with high intensity technology involvement | Vianello 2013 | Avoidance of hospital admission | Neuromuscular Disease Patients with severe respiratory tract infection | Hospital at home | Intermediate intensity - telephone, remote monitoring | Hybrid team of hospital-based health professionals and community nurse | Speciality treatment (respiratory treatment, nurse and respiratory therapist home visiting for face to face activities) | NA | NA |
| ***General inpatient-level care, delivered by hospital- and community-based professionals, with low intensity technology involvement (1 intervention)*** |  |  |  |  |  |  |  |  |  |
| General inpatient-level care, delivered by hospital- and community-based professionals, with low intensity technology involvement | Skwarska 2000 | Early discharge | COPD exacerbations | Supported discharge | Low intensity | Hybrid (hospital specialists and nurses, community GPs) | General care services | NA | NA |
| ***Extended multidisciplinary inpatient-level care, delivered by hospital- or community-based professionals, with high intensity technology involvement (2 specific interventions)*** |  |  |  |  |  |  |  |  |  |
| Extended multidisciplinary inpatient-level care, delivered by hospital based health professionals, with high intensity technology involvement | Yao 2022 | Unspecified | Acute medical conditions | Advanced Care at Home | High intensity - fully technology enabled | Hospital-based health professionals (including virtual ward staff for virtual care) | Multidisciplinary care (virtual round, face to face activities, post acute care) | Patient empowerment | NA |
| Extended multidisciplinary inpatient-level care, delivered by hospital-based health professionals, with high intensity technology involvement | Levine 2018, 2019 | Avoidance of hospital admission | Acute medical conditions | Home Hospital | Intermediate intensity - telephone/ video conferencing, remote monitoring | Hospital based health professionals including AHPs (MDT, consultant supervised) | Multidisciplinary care (nurse and physician home visiting for face to face activities) | Non-patient empowerment | NA |
| ***Extended multidisciplinary inpatient-level care, delivered by hospital- and community-based professionals, with low intensity technology involvement (9 specific interventions)*** |  |  |  |  |  |  |  |  |  |
| Extended multidisciplinary inpatient-level care, delivered by hospital- and community-based professionals, with low intensity technology involvement | Aimonino 2002; Aimonino Ricauda 2008; Ricauda et al 2004, Ahrens 2004, Ricauda 2005; Tibaldi 2009; Tibaldi 2013 | Avoidance of hospital admission | Advanced dementia, cognitively impaired older patients | Geriatric home hospitalisation service | Low intensity - telephone | Hybrid team of hospital based staff and AHPs (MDTs involving GP) | Multidisciplinary care (nurse and physician home visiting for face to face activities, rehabilitation) | Patient empowerment | NA |
| Extended multidisciplinary inpatient-level care, delivered by hospital- and community-based professionals, with low intensity technology involvement | Cai 2017 | Avoidance of hospital admission | General conditions | Hospital at home | Low intensity - telephone | Hybrid team of hospital-based staff and community staff (geriatrician supervised, community pharmacist involved) | Multidisciplinary care (nurse and physician home visiting for face to face activities) | Non-patient empowerment | Electronic record system |
| Extended multidisciplinary inpatient-level care, delivered by hospital- and community-based professionals, with low intensity technology involvement | Cai 2018 | Avoidance of hospital admission | General conditions | Cincinnati hospital at home | Low intensity - telephone | Hybrid team of hospital based staff, AHPs, and community staff (geriatrician supervised MDT, community pharmacist involved) | Multidisciplinary care (nurse and physician home visiting for face to face activities) | Non-patient empowerment | NA |
| Extended multidisciplinary inpatient-level care, delivered by hospital- and community-based professionals, with low intensity technology involvement | Harris 2005 | Avoidance of hospital admission, Early discharge | Acute medical conditions | Hospital at home | Low intensity - telephone | Hybrid team of hospital-based staff, AHPs, and GP (nurse led MDTs, geriatricians supervised, GP involved) | Multidisciplinary care (home support with live-in home carer for face to face activities, rehabilitation) | Non-patient empowerment | NA |
| Extended multidisciplinary inpatient-level care, delivered by hospital- and community-based professionals, with low intensity technology involvement | Leff 2005 | Avoidance of hospital admission | Acute medical conditions | Hospital at home | Low intensity - communication devices | Hybrid team of hospital-based health professionals and community team and home care agency | Multidisciplinary care (nurse and physician home visiting for face to face activities) | NA | NA |
| Extended multidisciplinary inpatient-level care, delivered by hospital- and community-based professionals, with low intensity technology involvement | Shepperd 1998 | Avoidance of hospital admission | Medical and surgical conditions | Hospital at home | Low intensity - telephone | Hybrid team of hospital staff (nurse), AHPs and GP (MDT) | Multidisciplinary care (nurse home visiting for face to face activities, nursing care dominated, rehabilitation) | NA | NA |
| Extended multidisciplinary inpatient-level care, delivered by hospital- and community-based professionals, with low intensity technology involvement | Wilson 1998, Wilson 1999, Wilson 2002, Jones 1999 | Avoidance of hospital admission | Acute medical conditions | Leicester hospital at home scheme | Low intensity - telephone | Hybrid team of hospital-based staff, AHPs, and community team (nurse led MDT, GP and community team involved) | Multidisciplinary care (community team home visiting for face to face activities, rehabilitation) | Non-patient empowerment | NA |
| Extended multidisciplinary inpatient-level care, delivered by hospital- and community-based professionals, with low intensity technology involvement | Shepperd 2017; Shepperd 2021; Singh 2022, ISRCTN60477865 | Avoidance of hospital admission | Older people with markers of frailty or prior dependence | Geriatrician-led admission avoidance hospital at home | Low intensity – virtual ward rounds using technologies that were not specified | Hybrid team of hospital based staff, AHPs and community team (geriatrician-led MDT) | Multidisciplinary care (virtual round, face to face activities) | NA | NA |
| Extended multidisciplinary inpatient-level care, delivered by hospital- and community-based professionals, with low intensity technology involvement | Tsiachristas 2019 | Avoidance of hospital admission | NA | Geriatrician-led admission avoidance hospital-at-home services | Low intensity – virtual ward rounds using technologies that were not specified | Hybrid team of hospital based staff and community team (geriatrician-led MDT) | Multidisciplinary care (geriatricians and MDT home visiting, rehabilitation) | NA | NA |
| ***Extended multidisciplinary inpatient-level care, delivered by hospital- or community-based health professionals, with low intensity technology involvement (4 interventions)*** |  |  |  |  |  |  |  |  |  |
| Extended multidisciplinary inpatient-level care, delivered by hospital based health professionals, with low intensity technology involvement | Echevarria 2018 (ISRCTN29082260) | Early discharge | COPD acute exacerbations | Hospital at home | Low intensity - telephone | Hospital based health professionals including AHPs (respiratory consultant supervised) | Multidisciplinary care (acute COPD services, nurse home visiting for face to face activities) | Non-patient empowerment | NA |
| Extended multidisciplinary inpatient-level care, delivered by hospital based health professionals, with low intensity technology involvement | Federman 2018, Augustine 2021; Saenger 2022 | Avoidance of hospital admission | Acute medical conditions | Hospital at home | Low intensity - telephone/ video conferencing | Hospital based health professionals including AHPs (MDT) | Multidisciplinary care (nurse and physician home visiting for face to face activities, post acute care) | Patient empowerment | NA |
| Extended multidisciplinary inpatient-level care, delivered by hospital based health professionals, with low intensity technology involvement | Jester 2003 | Early discharge | Orthopaedic surgical patients | Early discharge to hospital at home on the fourth postoperative day | Low intensity - telephone | Hospital-based health professionals including AHPs (orthopaedic consultant surgeon supervised) | Multidisciplinary care (post operative rehabilitation, nurse or physiotherapist home visiting for face to face activities) | Non-patient empowerment | NA |
| Extended multidisciplinary inpatient-level care, delivered by hospital based health professionals, with low intensity technology involvement | Pouw 2018, NTR6581 | Avoidance of hospital admission | Advanced dementia, cognitively impaired older patients | Hospital at home | Low intensity - telephone | Hospital based health professionals including AHPs (hospital specialist supervised) | Multidisciplinary care (nurse and physician home visiting for face to face activities) | Patient empowerment | NA |
| ***Interventions that have no detail of technology, workforce, or care pathway, or the three components (22 specific interventions)*** |  |  |  |  |  |  |  |  |  |
| General inpatient-level care, delivered by hospital- and community-based professionals | Davies 2000 | Avoidance of hospital admission | COPD acute exacerbations | Home care | NA | Hybrid team of hospital visiting nurses and district nurses (nurse dominated) | Speciality treatment (COPD treatment, nurse home visiting) | NA | NA |
| Extended multidisciplinary inpatient-level care, delivered by community based health professionals | O'Cathain 1994 | Early discharge | Orthopaedic surgical patients | Hospital at home | NA | Community-based health professionals including AHPs (community team dominated MDT, GP supervised) | Multidisciplinary care (community team home visiting) | NA | Electronic record system |
| General inpatient-level care, delivered by community based health professionals | Adler 1978 | Early discharge | Inguinal hernia or varicose veins (surgeries) | Short stay (early discharge) | NA | Community based health professionals (GP, community nurses, face to face activities) | General care services | NA | NA |
| General inpatient-level care, delivered by hospital based health professionals | Booth 2004 | Early discharge | Coronary artery bypass grafting | Early discharge | NA | Hospital based health professionals (face to face activities) | General care services | NA | NA |
| Extended multidisciplinary inpatient-level care, delivered by hospital- and community-based professionals | Caplan 1999; Caplan 2005; Board 2000 | Avoidance of hospital admission | Elderly people admitted to ED for acute medical conditions | Hospital in the home | NA | Hybrid (community GPs and hospital doctors and nurses, AHPs) | Multidisciplinary care | NA | NA |
| Extended multidisciplinary inpatient-level care, delivered by hospital- and community-based professionals | Kalra 2000; Patel 2003 | Avoidance of hospital admission | Acute stroke | Home stroke care | NA | Hybrid (hospital specialists and nurses, community GPs and nurses, AHPs) | Multidisciplinary care | NA | NA |
| Extended multidisciplinary inpatient-level care, delivered by hospital- and community-based professionals | Escartin 2017 | Avoidance of hospital admission, Early discharge | Acute calculous cholecystitis | Hospital at home | NA | Hybrid team of hospital ward health professionals and community team (surgeon led) | Multidisciplinary care (nurse home visiting for face to face activities) | Non-patient empowerment | NA |
| Extended multidisciplinary inpatient-level care, delivered by hospital- and community-based professionals | Hensher 1996 | Early discharge | Orthopaedic surgical patients | Hospital at home | NA | Hybrid team of hospital staff (nurses), AHPs, and community team (MDT) | Multidisciplinary care (face to face activities, post acute care) | NA | NA |
| Extended multidisciplinary inpatient-level care, delivered by a hospital based health professionals | ISRCTN36662318 | Early discharge | Acute medical conditions | Hospital at home | NA | Hospital based health professionals including AHPs | Multidisciplinary care (face to face activities, rehabilitation) | Non-patient empowerment | NA |
| Extended multidisciplinary inpatient-level care, delivered by hospital- and community-based professionals | Richards 1998, Coast 1998, Gunnell 2000 | Early discharge | Hospitalised but medically stable elderly patients | Hospital at home | NA | Hybrid team of hospital staff, AHPs, and community team (MDT) | Multidisciplinary care (face to face activities, rehabilitation) | NA | NA |
| Extended multidisciplinary inpatient-level care, delivered by a team with no further description | Rousseau 2019 | Unspecified | Acute respiratory conditions | Hospital at home | NA | A team with no further description | Multidisciplinary care (post acute care) | NA | NA |
| General inpatient-level care | Soones 2016 | Unspecified | Acute medical conditions | Mobile Acute Care Team | NA | NA | Acute and post acute care (nurse and physician home visiting) | NA | NA |
| Care services, delivered by hospital- and community-based professionals, with low intensity technology involvement | ISRCTN36101176 | Unspecified | COPD acute exacerbations | Hospital-at-home | Low intensity - telephone | Hybrid team of hospital based staff and community team | NA (face to face activities) | NA | NA |
| Specific treatment | NCT03490084 | Early discharge | Multiple myeloma | One day hospitalisation and 3 weekly chemotherapy hospital-at-home | NA | NA | Specific treatments (chemotherapy at home) | NA | NA |
| Specific treatments | Esmond 2006 | Avoidance of hospital admission | CF pulmonary exacerbations | Home IV antibiotics | NA | NA | IV antibiotics | Patient empowerment | NA |
| Specific treatments | Hatziagorou 2015 | Unspecified | CF pulmonary exacerbations | Home IV antibiotics | NA | NA | IV antibiotics | NA | NA |
| Specific treatments | Sequerios 2013 | Unspecified | CF pulmonary exacerbations | Hospital at home | NA | NA | IV antibiotics | NA | NA |
| Components-unspecified interventions | Aibar 2013 | Avoidance of hospital admission | Cancer | Hospital at home | NA | NA | NA | NA | NA |
| Components-unspecified interventions | Cai 2021 | Early discharge | General conditions | Transfer component of a Veterans Affairs Hospital in Home programme | NA | NA | NA | NA | NA |
| Components-unspecified interventions | Gruss 2013 | Unspecified | Acute respiratory conditions | Hospital at home | NA | NA | NA | NA | NA |
| Components-unspecified interventions | Morgan 2019 | Unspecified | COPD acute exacerbations | Hospital at home | NA | NA | NA | NA | NA |
| Components-unspecified interventions | Oterino-de-la-Fuente 1998 | Unspecified | General acute and non-acute conditions | Hospital at home | NA | NA | NA | NA | NA |

**Table S6. Summary of findings tables for individual outcomes**

| 1. **Summary of findings for the mortality outcome** | | | | | | |
| --- | --- | --- | --- | --- | --- | --- |
| **Outcomes** | **Anticipated absolute effects^*^ (95% CI)** | | **Relative effect (95% CI)** | **№ of participants (studies)** | **Certainty of the evidence (GRADE)** | **Comments** |
|  | **Risk with hospital based inpatient care** | **Risk with any groups of inpatient-level care at home interventions** |  |  |  |  |
| **Group 1. General inpatient-level care, delivered by hospital- or community-based professionals, with high intensity technology involvement** |  |  |  |  |  |  |
| Mortality (RCTs) | 143 per 1,000 | **103 per 1,000** (26 to 421) | **RR 0.72** (0.18 to 2.95) | 57 (1 RCT) | ⨁⨁◯◯ Low ^a^ | It is uncertain if there is a difference between general inpatient care, delivered by hospital- or community-based professionals, with high intensity technology involvement and hospital based inpatient care in mortality rates. |
| Mortality (non-randomised studies) | 39 per 1,000 | **7 per 1,000** (1 to 44) | **RR 0.18** (0.03 to 1.12) | 3573 (3 observational studies) | ⨁◯◯◯ Very low ^b,c^ |  |
| **Group 2. General inpatient-level care, delivered by hospital- or community-based professionals, with low intensity technology involvement** |  |  |  |  |  |  |
| Mortality (RCTs) | 52 per 1,000 | **30 per 1,000** (10 to 89) | **RR 0.58** (0.20 to 1.71) | 345 (4 RCTs) | ⨁⨁◯◯ Low ^a^ | It is uncertain if there is a difference between general inpatient care, delivered by hospital- or community-based professionals, with low intensity technology involvement and hospital based inpatient care in mortality rates. |
| Mortality (non-randomised studies) | 59 per 1,000 | **109 per 1,000** (38 to 315) | **RR 1.84** (0.64 to 5.31) | 216 (3 observational studies) | ⨁◯◯◯ Very low ^a,b^ |  |
| **Group 3. General inpatient-level care, delivered by hospital- and community-based professionals, with high intensity technology involvement** |  |  |  |  |  |  |
| Mortality (RCTs) | 148 per 1,000 | **116 per 1,000** (28 to 467) | **RR 0.78** (0.19 to 3.15) | 53 (1 RCT) | ⨁⨁◯◯ Low ^a^ | It is uncertain if there is a difference between general inpatient care, delivered by hospital- and community-based professionals, with high intensity technology involvement and hospital based inpatient care in mortality rates. |
| Mortality (non-randomised studies) | 19 per 1,000 | **20 per 1,000** (1 to 311) | **RR 1.04** (0.07 to 16.18) | 102 (1 observational study) | ⨁◯◯◯ Very low ^a,d^ |  |
| **Group 4. General inpatient-level care, delivered by hospital- and community-based professionals, with low intensity technology involvement** |  |  |  |  |  |  |
| Mortality (RCTs) | 113 per 1,000 | **33 per 1,000** (10 to 107) | **RR 0.29** (0.09 to 0.95) | 184 (1 RCT) | ⨁⨁◯◯ Low ^a^ | General inpatient care, delivered by hospital- and community-based professionals, with low intensity technology involvement may have a lower mortality rate than hospital based inpatient care. |
| **Group 5. Extended multidisciplinary inpatient-level care, delivered by hospital-based health professionals, with intermediate or high intensity technology involvement** |  |  |  |  |  |  |
| None |  |  |  |  |  |  |
| **Group 6. Extended multidisciplinary inpatient-level care, delivered by hospital- or community-based professionals, with low intensity technology involvement** |  |  |  |  |  |  |
| Mortality (RCTs) | 17 per 1,000 | **17 per 1,000** (1 to 260) | **RR 0.97** (0.06 to 15.09) | 118 (1 RCT) | ⨁⨁◯◯ Low ^a^ | It is uncertain if there is a difference between extended multidisciplinary services, delivered by hospital- or community-based professionals, with low intensity technology involvement and hospital based inpatient care in mortality rates. |
| Mortality (non-randomised studies) | 0 per 1,000 | **0 per 1,000** (0 to 0) | **RR 2.16** (0.09 to 52.74) | 507 (1 observational study) | ⨁◯◯◯ Very low ^a,d^ |  |
| **Group 7. Extended multidisciplinary inpatient-level care, delivered by hospital- and community-based professionals, with low intensity technology** |  |  |  |  |  |  |
| Mortality (RCTs) | 107 per 1,000 | **103 per 1,000** (85 to 124) | **RR 0.96** (0.79 to 1.16) | 2727 (7 RCTs) | ⨁⨁⨁◯ Moderate ^c^ | There is probably no difference between extended multidisciplinary services, delivered by hospital- and community-based professionals, with low intensity technology involvement and hospital based inpatient care in mortality rates. |
| Mortality (non-randomised studies) | 65 per 1,000 | **30 per 1,000** (16 to 56) | **RR 0.47** (0.25 to 0.86) | 24305 (4 observational studies) | ⨁◯◯◯ Very low ^b^ |  |

| 1. **Summary of findings for the hospital readmission outcome** | | | | | | |
| --- | --- | --- | --- | --- | --- | --- |
| Outcomes | **Anticipated absolute effects^*^** (95% CI) | | Relative effect (95% CI) | № of participants (studies) | Certainty of the evidence (GRADE) | Comments |
|  | **Risk with hospital based inpatient care** | **Risk with any groups of inpatient-level care at home interventions** |  |  |  |  |
| **Group 1. General inpatient-level care, delivered by hospital- or community-based professionals, with high intensity technology involvement** |  |  |  |  |  |  |
| Number of hospital readmission events (non-randomised studies) | 105 per 1,000 | **69 per 1,000** (23 to 203) | **RR 0.65** (0.22 to 1.93) | 3002 (2 observational studies) | ⨁◯◯◯ Very low ^a,b,e^ | It is uncertain in the number of hospital readmission events between general inpatient care, delivered by hospital- or community-based professionals, with high intensity technology involvement and hospital based inpatient care. |
| **Group 2. General inpatient-level care, delivered by hospital- or community-based professionals, with low intensity technology involvement** |  |  |  |  |  |  |
| Number of hospital readmission events (RCTs) | 156 per 1,000 | **161 per 1,000** (123 to 209) | **RR 1.03** (0.79 to 1.34) | 1022 (7 RCTs) | ⨁⨁◯◯ Low ^a^ | There may be no difference in the number of hospital readmission events between general inpatient care, delivered by hospital- or community-based professionals, with low intensity technology involvement and hospital based inpatient care. However there are some uncertainties around this as the wide confidence interval includes risk of benefits and harms |
| Number of hospital readmission events (non-randomised studies) | 239 per 1,000 | **225 per 1,000** (113 to 450) | **RR 0.94** (0.47 to 1.88) | 126 (2 observational studies) | ⨁◯◯◯ Very low ^a,b^ |  |
| **Group 3. General inpatient-level care, delivered by hospital- and community-based professionals, with high intensity technology involvement** |  |  |  |  |  |  |
| Number of hospital readmission events (non-randomised studies) | 750 per 1,000 | **277 per 1,000** (173 to 450) | **RR 0.37** (0.23 to 0.60) | 102 (1 observational study) | ⨁⨁◯◯ Low ^a,d,f^ | General inpatient care, delivered by hospital- and community-based professionals, with high intensity technology involvement may have a lower number of hospital readmission events than hospital based inpatient care. |
| **Group 4. General inpatient-level care, delivered by hospital- and community-based professionals, with low intensity technology involvement** |  |  |  |  |  |  |
| Number of hospital readmission events (RCTs) | 339 per 1,000 | **220 per 1,000** (135 to 359) | **RR 0.65** (0.40 to 1.06) | 184 (1 RCT) | ⨁⨁◯◯ Low ^a^ | General inpatient care, delivered by hospital- and community-based professionals, with low high intensity technology involvement may have a lower number of hospital readmission events than hospital based inpatient care. |
| **Group 5. Extended multidisciplinary inpatient-level care, delivered by hospital- or community-based professionals, with high intensity technology involvement** |  |  |  |  |  |  |
| Number of hospital readmission events (RCTs) | 254 per 1,000 | **76 per 1,000** (28 to 219) | **RR 0.30** (0.11 to 0.86) | 111 (2 RCTs) | ⨁⨁◯◯ Low ^a^ | Extended multidisciplinary services, delivered by hospital- or community-based professionals, with high intensity technology involvement may have a lower number of hospital readmission events than hospital based inpatient care. |
| **Group 6. Extended multidisciplinary inpatient-level care, delivered by hospital- or community-based professionals, with low intensity technology involvement** |  |  |  |  |  |  |
| Number of hospital readmission events (RCTs) | 397 per 1,000 | **365 per 1,000** (230 to 579) | **RR 0.92** (0.58 to 1.46) | 118 (1 RCT) | ⨁⨁◯◯ Low ^a^ | There may be no difference in the number of hospital readmission events between extended multidisciplinary services, delivered by hospital- or community-based professionals, with low intensity technology involvement and hospital based inpatient care. However there are some uncertainties around this as the wide confidence interval includes risk of benefits and harms |
| Number of hospital readmission events (non-randomised studies) | 151 per 1,000 | **85 per 1,000** (51 to 139) | **RR 0.56** (0.34 to 0.92) | 507 (1 observational study) | ⨁⨁◯◯ Low ^d,g^ |  |
| **Group 7. Extended multidisciplinary inpatient-level care, delivered by hospital- and community-based professionals, with low intensity technology** |  |  |  |  |  |  |
| Number of hospital readmission events (RCTs) | 309 per 1,000 | **291 per 1,000** (214 to 396) | **RR 0.94** (0.69 to 1.28) | 2257 (6 RCTs) | ⨁⨁◯◯ Low ^g,h^ | There may be no difference in the number of hospital readmission events between extended multidisciplinary services, delivered by hospital- and community-based professionals, with low intensity technology involvement and hospital based inpatient care. However there are some uncertainties around this as the wide confidence interval includes risk of benefits and harms |
| Number of hospital readmission events (non-randomised studies) | 208 per 1,000 | **188 per 1,000** (150 to 236) | **RR 0.90** (0.72 to 1.13) | 1695 (3 observational studies) | ⨁◯◯◯ Very low ^b,g^ |  |

| 1. **Summary of findings for the length of stay outcome** | | | | | | |
| --- | --- | --- | --- | --- | --- | --- |
| Outcomes | **Anticipated absolute effects^*^** (95% CI) | | Relative effect (95% CI) | № of participants (studies) | Certainty of the evidence (GRADE) | Comments |
|  | **Risk with hospital based inpatient care** | **Risk with any groups of inpatient-level care at home interventions** |  |  |  |  |
| **Group 1. General inpatient-level care, delivered by hospital- or community-based professionals, with high intensity technology involvement** |  |  |  |  |  |  |
| Length of stay (non-randomised studies) | The median length of stay (non-randomised studies) was **5** days | MD **1.07 days fewer** (2.04 fewer to 0.1 fewer) | - | 3573 (3 observational studies) | ⨁◯◯◯ Very low ^b,e^ | It is uncertain in the length of stays between general inpatient care, delivered by hospital- or community-based professionals, with high intensity technology involvement and hospital-based inpatient care. |
| **Group 2. General inpatient-level care, delivered by hospital- or community-based professionals, with low intensity technology involvement** |  |  |  |  |  |  |
| Length of stay in days (RCTs) | The median length of stay in days (RCTs) was **6.1** days | MD **0.29 days more** (2.56 fewer to 3.14 more) | - | 551 (3 RCTs) | ⨁⨁◯◯ Low ^c,h^ | There may be no difference in the length of stay in care between general inpatient care, delivered by hospital- or community-based professionals, with low intensity technology involvement and hospital-based inpatient care. However there are some uncertainties around this as the wide confidence interval includes risk of benefits and harms |
| Length of stay in days (non-randomised studies) | The mean length of stay in days (non-randomised studies) was **22.4** days | MD **5.3 days fewer** (7.72 fewer to 2.87 fewer) | - | 161 (2 observational studies) | ⨁◯◯◯ Very low ^a,b,f^ |  |
| **Group 3. General inpatient-level care, delivered by hospital- and community-based professionals, with intermediate or high intensity technology involvement** |  |  |  |  |  |  |
| None |  |  |  |  |  |  |
| **Group 4. General inpatient-level care, delivered by hospital- and community-based professionals, with low intensity technology involvement** |  |  |  |  |  |  |
| None |  |  |  |  |  |  |
| **Group 5. Extended multidisciplinary inpatient-level care, delivered by hospital- or community-based professionals, with high intensity technology involvement** |  |  |  |  |  |  |
| Length of stay (RCTs) | The mean length of stay (RCTs) was **3.5** days | MD **0.46 days more** (0.22 fewer to 1.14 more) | - | 111 (2 RCTs) | ⨁⨁◯◯ Low ^a^ | There may be no difference in the length of stay in care between extended multidisciplinary services, delivered by hospital- or community-based professionals, with high intensity technology involvement and hospital-based inpatient care. |
| **Group 6. Extended multidisciplinary inpatient-level care, delivered by hospital- or community-based professionals, with low intensity technology involvement** |  |  |  |  |  |  |
| Length of stay (RCTs) | The mean length of stay (RCTs) was **4.1** days | MD **2.9 days fewer** (4.2 fewer to 1.6 fewer) | - | 118 (1 RCT) | ⨁⨁◯◯ Low ^a^ | Extended multidisciplinary services, delivered by hospital- or community-based professionals, with low intensity technology involvement may have on average 2.9 days shorter (4.2 shorter to 1.6 shorter) stays in care than hospital-based inpatient care. |
| Length of stay (non-randomised studies) | The mean length of stay (non-randomised studies) was **5.5** days | MD **2.3 days fewer** (2.82 fewer to 1.78 fewer) | - | 507 (1 observational study) | ⨁⨁⨁⨁ High ^d,f^ |  |
| **Group 7. Extended multidisciplinary inpatient-level care, delivered by hospital- and community-based professionals, with low intensity technology** |  |  |  |  |  |  |
| Length of stay (RCTs) | The median length of stay (RCTs) was **10** days | MD **4.85 days more** (1.8 more to 7.91 more) | - | 1694 (6 RCTs) | ⨁⨁◯◯ Low ^g,h^ | Extended multidisciplinary services, delivered by hospital- and community-based professionals, with low intensity technology involvement may have on average 4.85 days longer (1.8 to 7.91 longer) stays in care than hospital-based inpatient care. |
| Length of stay (non-randomised studies) | The mean length of stay (non-randomised studies) was **4.9** days | MD **1.7 days fewer** (2.91 fewer to 0.49 fewer) | - | 455 (1 observational study) | ⨁◯◯◯ Very low ^b,g^ |  |

**Notes:**

a. Downgraded twice for imprecision as the optimal information size is unmet, and there are very wide confidence interval and/or small sample sizes.

b. Downgraded three times for risk of bias, as per GRADE guidance (1), due to extremely serious limitations in most non-randomised studies included.

c. Downgraded once for imprecision as the optimal information size is met but there is very wide confidence interval.

d. Downgraded once for risk of bias as the non-randomised studies included are at moderate risk of bias.

e. Downgraded twice for substantial inconsistency that can only be explained partly.

f. Upgraded once for the large effect size as there are more than 2 days reduction in length of stay or RR < 0.50.

g. Downgraded once for imprecision ad the optimal information size is unmet but the numbers of events are large.

h. Downgraded once for moderate inconsistency that can only be explained partly.

(1) Piggott T, Morgan RL, Cuello-Garcia CA, Santesso N, Mustafa RA, Meerpohl JJ, Schünemann HJ, GRADE Working Group. Grading of Recommendations Assessment, Development, and Evaluations (GRADE) notes: extremely serious, GRADE's terminology for rating down by three levels. Journal of clinical epidemiology. 2020 Apr 1;120:116-20.

**Text 3. Results of cost and cost effectiveness analyses**

**Part 1. Cost effectiveness results**

Two studies reported the relative cost‐effectiveness of inpatient-level care at home interventions in comparison with hospital-based inpatient care. Both evaluations were conducted in the UK context, and their results consistently suggested that inpatient-level care at home may be more cost effective than inpatient care.

- A UK trial-based ***cost utility analysis*** of 118 participants with COPD exacerbations (with the trial at unclear risk of bias) suggested that mean 90-day costs were £1016 lower in hospital at home that we defined as extended multidisciplinary inpatient-level care, delivered by hospital- or community-based professionals, with low intensity technology involvement, but the one-sided 95% CI crossed the non-inferiority limit of £150 (CI −2343 to 312). (41) Savings were primarily due to reduced hospital bed days (1, IQR 1–7 days for hospital at home; 5, IQR 2–12 days for the control group; P=0.001). For the model extended multidisciplinary inpatient-level care, delivered by hospital- or community-based professionals, with low intensity technology involvement, its probability of being cost-effective was 90% at the NICE threshold of £30000 per QALY for patients with COPD exacerbation when quality-adjusted life years was considered in the analysis. Hospital at home was both cheaper and more effective for most patients with COPD exacerbations treated (74% probability).
- Another UK trial-based ***cost effectiveness and cost utility analyses*** of 457 stroke participants (low risk of bias) estimated incremental cost-effectiveness ratios (ICERs), representing additional cost per additional percentage point of deaths or institutionalisations avoided, and incremental cost per additional QALY gained. This evaluation suggested that mean healthcare and social care costs over 12 months were £11 450 for (hospital-based) stroke unit, £9527 for (hospital-based) stroke team, and £6840 for domiciliary care. (We classified the domiciliary care model to the model group: extended multidisciplinary inpatient-level care, delivered by hospital- and community-based professionals). Inclusion of informal care increased costs considerably. The (hospital-based) stroke team was dominated by domiciliary care, meaning that using (hospital-based) stroke team was more expensive and less effective. When informal care was excluded, the incremental cost-effectiveness ratio per percentage point in deaths or institutionalisations avoided in the first year was £496 for the (hospital-based) stroke unit over domiciliary care according to cost effectiveness analysis. Incremental cost per quality-adjusted life year quality-adjusted life year gained was £64 097 between the (hospital-based) stroke unit and domiciliary care according to cost utility analysis. (10, 11) At a willingness to pay of £30 000 per additional QALY, the probability of the interventions being cost effective is at 29% for hospital-based stroke unit and stroke team and at 42% for domiciliary care.

**Part 2. Cost results**

A total of 31 studies did not report comparative relative cost effectiveness results, of which 28 (31; 90.3%) suggest that use of inpatient-level care at home interventions cost equivalently or less than use of hospital-based inpatient care. Only three studies reported a higher cost for use of inpatient-level care at home interventions (Sartain 2002; Harris 2005; Shepperd 1998).

| **Studies** | **Summary results of cost or cost effectiveness** |
| --- | --- |
| ***RCTs*** |  |
| Aimonino Ricauda 2008 | Cost per patient per day: $101.4 (SD 61.3) in intervention patients vs $151.7 (96.4) in control patients, P=0.002 |
| Adler 1978 | The savings to the statutory services of discharging patients early were estimated at £25.72 per patient |
| Booth 2004 | The total costs at 12 week follow up were higher in the conventional hospital care group compared to early discharge (£6381 v £6127) |
| Caplan 2000 | Costs per episode (AUD): lower costs per separation ($1764, 95% $1416 to $2111, n=50) in the inpatient care at home group than the control inpatient care group ($3614, CI 95% $2881.37 to $4347.27, n=47) (p < 0.0001, Mann-Whitney U - Wilcoxon Rank Sum) |
| Campbell 2001 | The cost consequence analysis using a cohort design (51 participants, with critical risk of bias) in UK medical and orthopaedic surgical people suggested that the model (general inpatient-level care, delivered by hospital- or community-based health professionals, with low intensity technology involvement) was more cost saving compared with hospital-based inpatient care, considering both the treatment phase and the three-month follow-up, with the total mean cost differential per patient (i.e. cost saving) at £1884. The study researchers used a discrete event simulation model which only considered hospital readmission events. They compared between the two care models in terms of their costs and suggested rates of hospital re-admissions after discharge were similar in the two groups. The evidence is uncertain due to the small sample size and methodological limitations of the cohort study used. |
| Bagust 2002, Sartain 2002, ISRCTN11421664 | Direct costs borne by families: £23.31 v £13.76, p = 0.001; and total NHS costs per patient: £870 v £741. Paediatric HAH schemes are unlikely to reduce NHS costs and do not increase privately borne costs |
| Harris 2005 | The average total cost per patient: NZ$6524 for the intervention group vs NZ$3525 for the control group (P<0.0001). The cost per patient day of service: NZ$570 vs NZ$538 |
| Hendricks 2011; Talcott 2011 | Home-based care costs on average $2,300 (in 2008 dollars) less per episode than continued inpatient care |
| Ince 2014 | Mean total charges in US dollars (USD): 138.57 (SD 72.87) in 42 intervention patients, 951.24 (SD 715.14) in 42 control patients |
| Levine 2019 | Mean unadjusted cost of the acute care episode was 41% lower for home patients than control patients (P < 0.001); the adjusted mean cost of the acute care episode was 38% (95% CI, 24% to 49%) lower for home patients than control patients (adjusted for sex, age, race/ethnicity, education, discharge diagnosis, and comorbid condition count) |
| Mendoza 2009 | Total cost per episode (in Euros): 2541 (SD 1334) in 37 intervention patients, 4502 (SD 2153) in 34 control patients |
| Ricauda et al 2004, Ahrens 2004, Ricauda 2005 | Cost per patient per day: $163.0 (SD 20.5) in 60 intervention patients, $275.6 (SD 27.7) in 60 control patients (P<0.001) |
| Richards 1998, Coast 1998, Gunnell 2000 | Mean cost (at 1995/6 values) per patient of resource use in 3 months (NHS or social services): 2515.71 for 160 intervention patients, 3291.99 for 81 control patients |
| Shepperd 1998 | No difference in total healthcare costs between groups for patients recovering from a hip or knee replacement, or elderly medical patients. Intervention significantly increased healthcare costs for patients recovering from a hysterectomy (ratio of geometrical means 1.15, 95% confidence interval 1.04 to 1.29, P = 0.009) and for those with chronic obstructive airways disease (Mann­ Whitney U test, P = 0.01). Interventions significantly increased general practitioners' costs for elderly medical patients (Mann­ Whitney U test, P < 0.01) and for those with chronic obstructive airways disease (P = 0.02). Patient and carer expenditure made up a small proportion of total costs |
| Shepperd 2017; Shepperd 2021; Singh 2022, ISRCTN60477865 | Adjusted for baseline covariates, the intervention group was less costly than admission to hospital from a health and social care perspective (mean -£2,265, 95%CI: -4,279 to -252), and remained less costly with the addition of informal care costs (mean difference -£2,840, 95%CI: -5,495 to -185). |
| Tibaldi 2009 | Mean total cost for each treated patient in euro: 1820.92 for 48 intervention patients, 2116.89 for 53 control patients |
| Vianello 2013 | Total and daily direct cost of patient healthcare: 542 (SD 258.5) vs 8,890 (10,992.7) (P<0.001) and 65.3 (18.6) vs 1,060 (592.5) (P<0.001), respectively. The district nurse service was the major cost for the subjects treated at home 320 (SD 118.5) |
| Wilson 1998, Wilson 1999, Wilson 2002, Jones 1999 | ITT analysis showed similar mean (median) costs per episode between the intervention group £2569 (£1655) and the control group £2881 (£2031), bootstrap mean difference - 305 (95% confidence interval - 1112 to 448). When analysis was restricted to those who accepted their allocated place of care, hospital at home was significantly cheaper—hospital at home £2557 (£1710), hospital ward £3660 (£2903), bootstrap mean difference - 1071 ( - 1843 to - 246). At three months the cost differences were sustained |
| ***Non-randomised studies*** |  |
| Cai 2018 | The intervention had on average lower total costs of services for treating an acute episode ($7,792) than the control group ($10,960) (p<0.001) |
| Cai 2021 | VA costs were 20% lower at 30 days (−$5910; 95%CI, −$13 049 to $1229) and 13% lower at 90 days (−$5793; 95%CI, −$19 179 to $7594) for hospital at home patients. Total VA and Medicare costs were 22% lower at 30 days (−$7002; 95% CI, −$14 314 to $309) and 12% lower at 90 days (−$6016; 95%CI, −$20 673 to $83641) for the transfer-hospital at home patients |
| Cryer 2012 | Mean hospital at home patient costs were 19 percent lower than mean hospital costs for comparison group patients. These savings were predominantly derived from lower average length-of-stay and lower use of clinical testing |
| Gruss 2013 | Control patients had higher costs ($U 31320 vs. $U 14915, p=0) |
| Hatziagorou 2015 | The patients treated at home had substantial economical savings (p<0.001) |
| Hensher 1996 | Cost of one day complete episode of care: Scheme 1=£220.10, Scheme 2=£98.61, Scheme 3=£129.49 in intervention patients, Scheme 1=£19164, Scheme 2=£105.51, Scheme 3=£132.84 in control patients |
| Herranz 2022 | Cost /patient in euros: 1126 (SD 226) for 137 intervention patients, 2346 (SD 519) for 137 control patients |
| Jester 2003 | Cost effectiveness analysis showed that the intervention group costed less, with the difference in cost per case between groups of -£652.04 per patient. This saving was achieved by a combination of reduced mean length of stay of 0.9 days and cheaper running costs of hospital at home |
| O'Cathain 1994 | Community Health Services quoted a charge of £400-£450 per patient compared with acute hospital charges of £770 for seven days in a hospital orthopaedic bed; these costs are charges, not actual costs |
| Oterino-de-la-Fuente 1998 | Mean cost of drugs, nursing materials, and diagnostic tests (CI) (in Spanish Pesetas): 10487 (7788-13187) for 148 intervention patients, 22556 (15084-30029) for 148 control patients |
| Rodriguez-Cerrillo 2013 | The cost of each patient treated at home was 1368 euros cheaper than that in the hospital (fewer staff and important reduction of maintenance costs |
| Saenger 2022 | Mean unadjusted total costs of the acute hospitalisation plus the 30-day post-acute period: $17,937 [$12,041] for the hospital at home group vs. $22,991 [25,423] for the control group. The matched unadjusted analysis suggested that average costs for the acute episode of care plus 30-day post-acute period were lower for hospital at home versus inpatient episodes of care (-$5116 [95% confidence interval -$10,262 to $30], p = 0.05). After adjusting for covariates, hospital at home costs remained lower (-$5977 [95% CI -$10,758 to -$1196], p = 0.01) |
| Tsiachristas 2019 | The cost during a hospital-at-home admission was on average lower than hospital admission in site 1 (mean difference -£2318; 95% CI: -2420 to -2217) and site 3 (mean difference -£1096; 95% CI: -£1398 to -£793), and slightly lower (mean difference -£153; 95% CI: -£277 to -£29) in site 2... After PSM and regression analysis, the healthcare cost for site 1 in hospital-at-home during the whole follow-up period (ie, during index admission and over 6 months after discharge from the index admission) was on average 18% lower (ratio of means: 0.82; 95% CI: 0.76 to 0.89) than admission to hospital |

**Table S7. Results of adverse event outcomes**

| **Studies** | **Adverse event outcomes and definitions** | **Interventions, rates (events/total) or no. of events** | **Control, rates (events/total) or no. of events** | **Comments** |
| --- | --- | --- | --- | --- |
| ***RCTs*** |  |  |  |  |
| Adler 1978 | Any complications up to 7 days | 12.8% (15/117) | 4.7% (5/107) |  |
| Caplan 2000 | Proportions of participants with adverse events | 11.8% (6/51) | 16.3% (8/49) |  |
| Hendricks 2011; Talcott 2011 | Major medical complications | 8.5% (4/47) | 7.6% (5/66) |  |
| Jakobsen 2013 | Returns to hospitals | 10.3% (3/29) | NA | 1 patient returned due to technical failure of wireless broadband technology in the home, 1 patient developed hyponatremia, and 1 patient had severe dyspnea and nebulizer failure |
| ***Non-randomised studies*** |  |  |  |  |
| Campbell 2001 | Relapsed during treatment phase | NA | 9.5% (2/21) | NA |
| Escartin 2017 | All grade of complications (that might not be related to hospital at home) | 6.1% (9/147) | 10.7% (82/768) | NA |
| Federman 2018, Augustine 2021 | Urinary catheter placement | 1.0% (3) | 4.4% (9) | Difference in event rates: −3.4% (95% CI −6.9% to −0.4%) P = 0.02 |
|  | Falls | 1.4% (4) | 0 | Difference in event rates: 1.4% (95% CI 0.04% to 2.7%), P = 0.09 |
|  | Nosocomial infections | 0 | 1.0% (2) | Difference in event rates: −1.0% (95%CI −2.3% to 0.4%), P = 0.09 |
| Hatziagorou 2015 | Rash | 5% (1/20) | No complications | NA |
| Jester 2003 | The incidence of postoperative complications (DVT, PE, dislocation, wound infection, pressure sores) | 2 Infection, 4 pressure ulcers | 2 Deep vein thrombosis, 6 Infection, 6 Pressure ulcers | Comparable incidence of postoperative complications |
| Leff 2005 | Critical complications | 0 (0/169) | 5.6% (16/286) |  |
| Oterino-de-la-Fuente 1998 | Complications measured by an adaptation of the Complication Screening Programme | 0.9% | 0.6% | Intervention group had a higher complication incidence |
