# Supplementary material for: Inpatient-level care at home delivered by virtual wards and hospital at home: a systematic review and meta-analysis of complex interventions and their components
Source: BMC Med. 2024 Apr 2;22:145. doi: 10.1186/s12916-024-03312-3 (PMC10986022; doi:10.1186/s12916-024-03312-3)
Supplement: Supplementary file 2 — Additional file 2: Fig. S1. Meta-analyses of RCT data by care models for mortality. Fig. S2. Meta-analyses of non-randomised data by care models for mortality. Fig. S3. Meta-analyses of RCT data by care models for hospital readmission. Fig. S4. Meta-analyses of non-randomised data by care models for hospital readmission. Fig. S5. Meta-analyses of RCT data by care models for the length of care stay in days. Fig. S6. Meta-analyses of non-randomised data by care models for the length of care stay in days. [file 12916_2024_3312_MOESM2_ESM.docx]

**
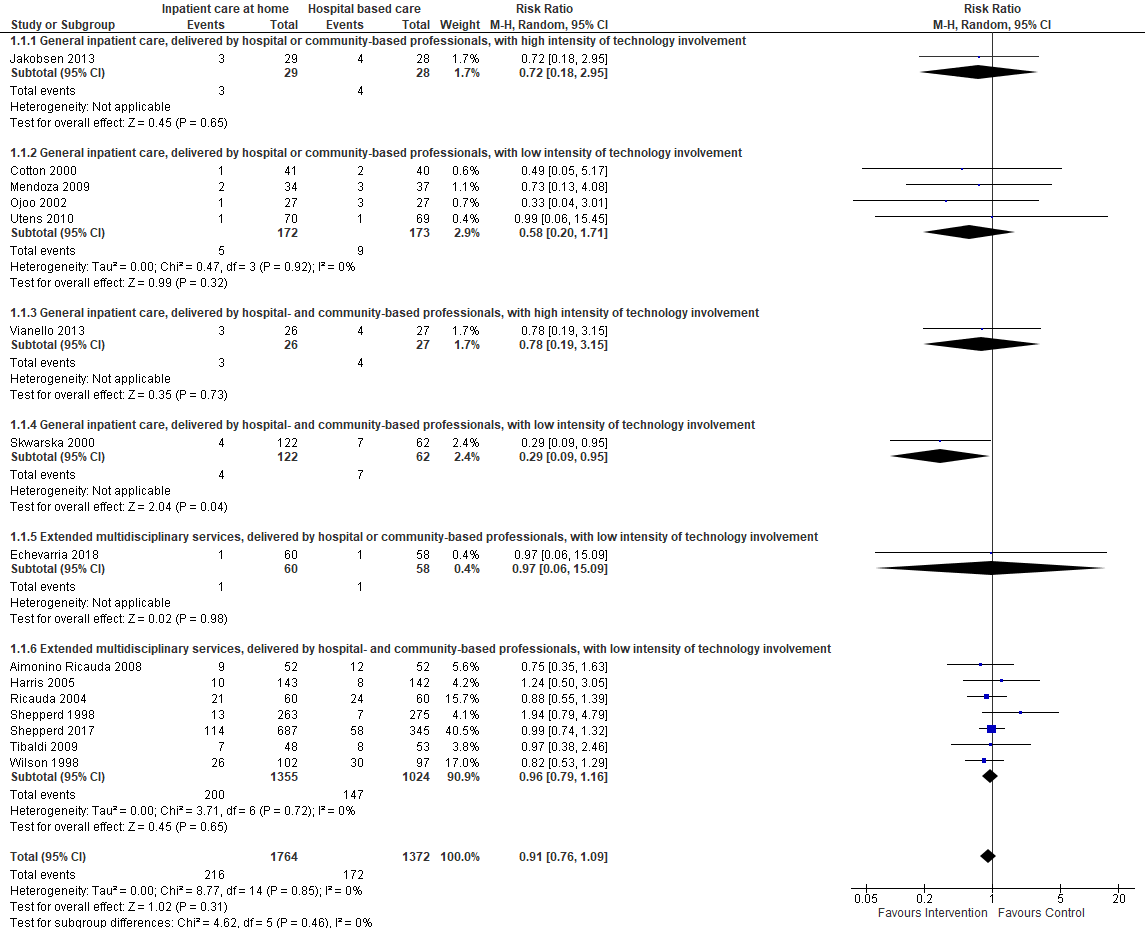
**

**Fig. S1. Meta-analyses of RCT data by care models for mortality**

**
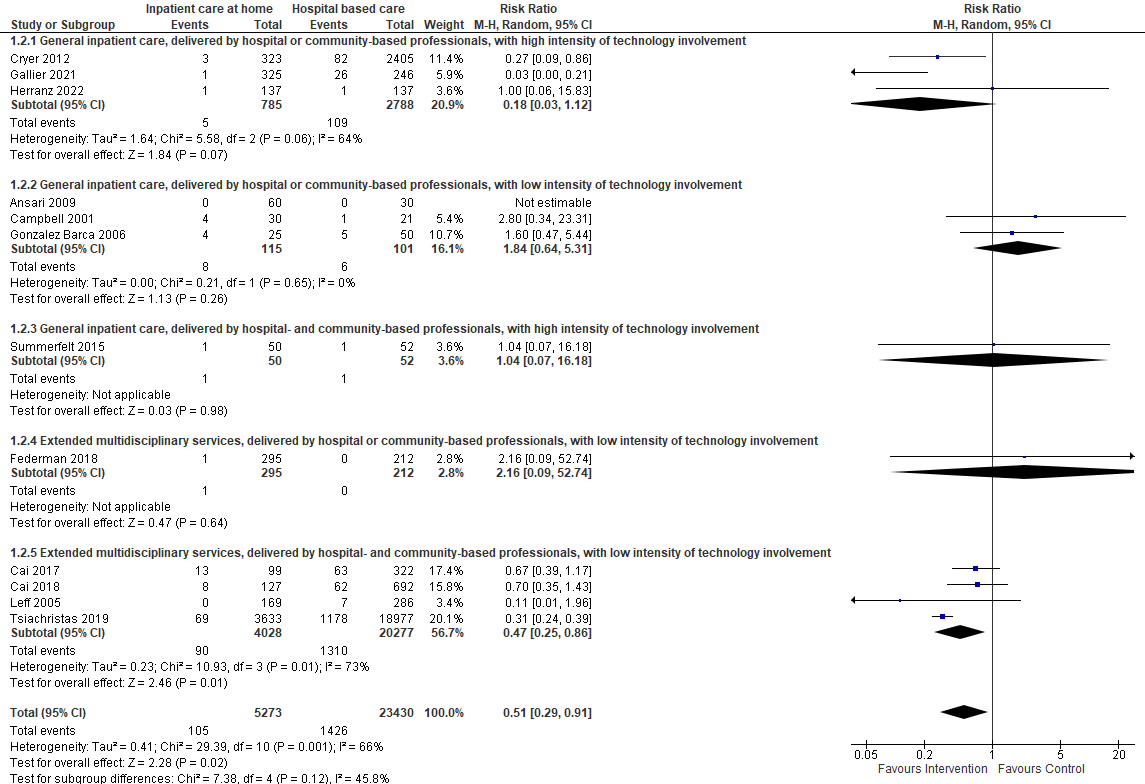
**

**Fig. S2. Meta-analyses of non-randomised data by care models for mortality**

**
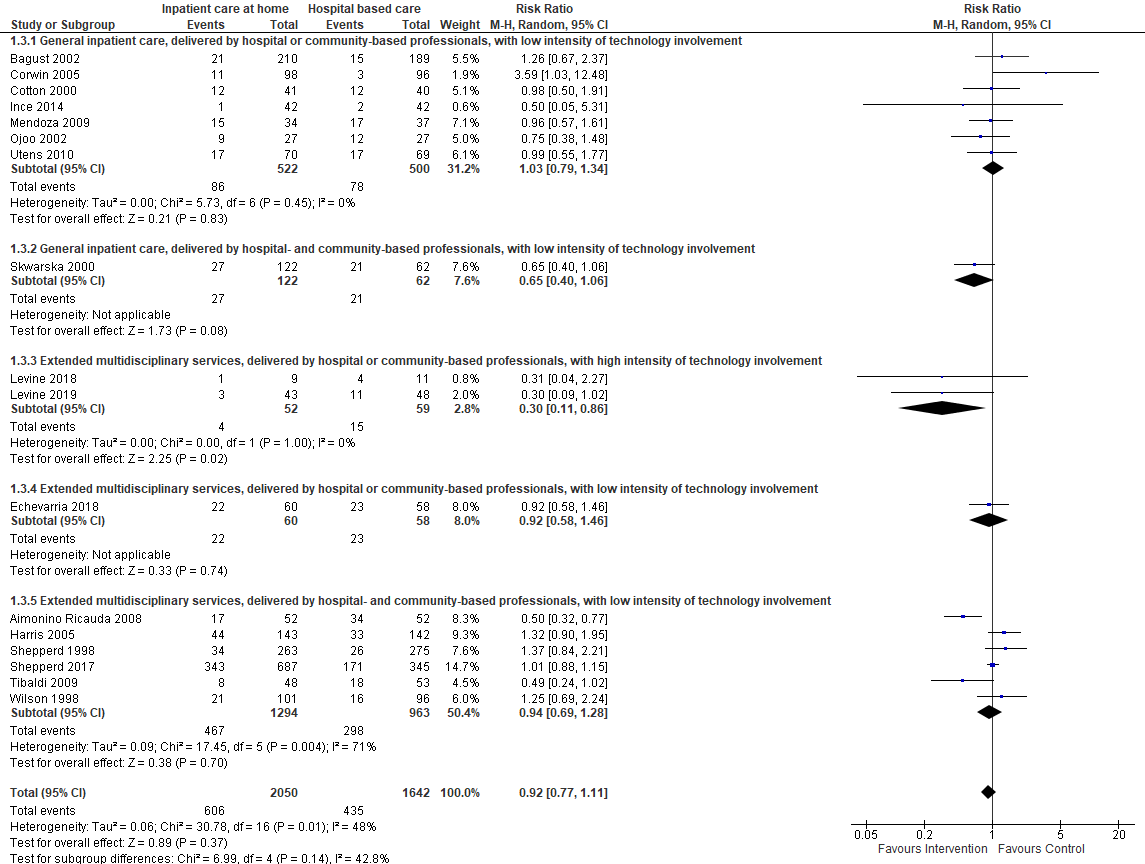
**

**Fig. S3. Meta-analyses of RCT data by care models for hospital readmission**

**
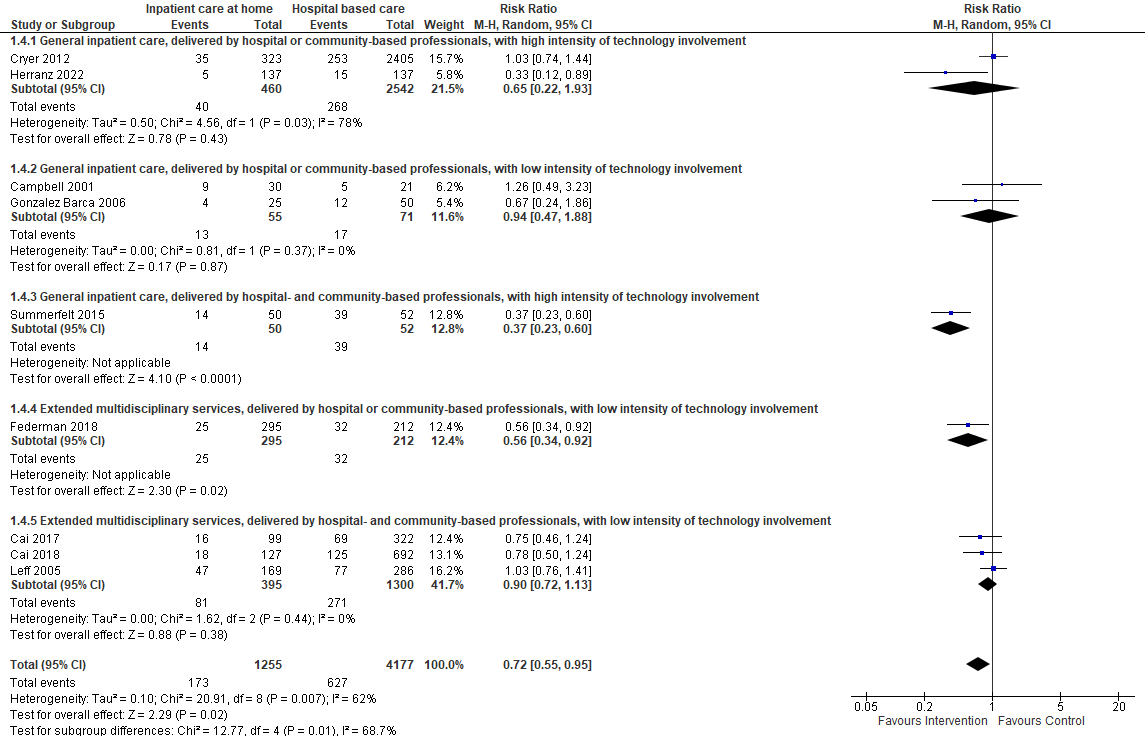
**

**Fig. S4. Meta-analyses of non-randomised data by care models for hospital readmission**

**
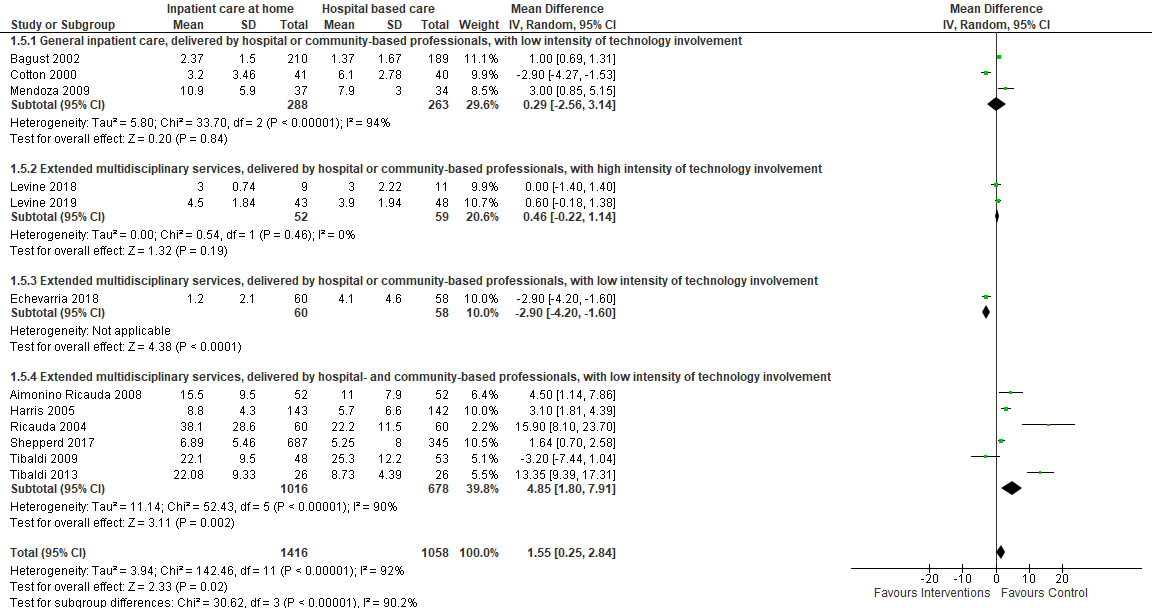
**

**Fig. S5. Meta-analyses of RCT data by care models for the length of care stay in days**

**
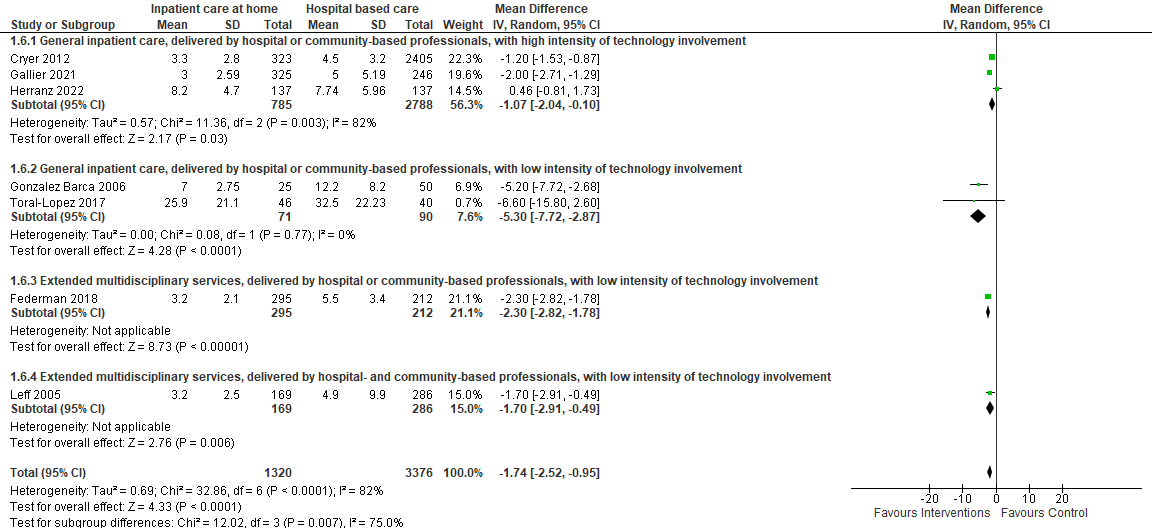
**

**Fig. S6. Meta-analyses of non-randomised data by care models for the length of care stay in days**
